# Effectiveness of a teaching unit on the willingness to consume insect-based food – An intervention study with adolescents from Germany

**DOI:** 10.3389/fnut.2022.889805

**Published:** 2022-10-05

**Authors:** Lena Szczepanski, Jacqueline Dupont, Fenja Schade, Henrike Hellberg, Milan Büscher, Florian Fiebelkorn

**Affiliations:** Biology Didactics, Department of Biology/Chemistry, Osnabrück University, Osnabrück, Germany

**Keywords:** entomophagy, edible insects, novel food, alternative protein, meat alternative, teenagers, schools, information

## Abstract

This study investigated the effect of a four-lesson teaching unit titled “Entomophagy and Sustainability” on the willingness of adolescents in Germany to consume insect-based food (*N* = 114; *M*_*Age*_ = 15.77 years; *SD*_*Age*_ = 1.12 years; female = 58.8%). The main aim of the study was to test whether the teaching unit can induce long-term changes in selected nutritional-psychological factors (food disgust, food neophobia, food technology neophobia), attitudes, knowledge, and the willingness to consume insect-based food. For this purpose, a paper-pencil questionnaire was conducted immediately before (pre-test), immediately after (post-test), and approximately six weeks after (follow-up test) the teaching unit. Although significant changes in food disgust, food neophobia, food technology neophobia, attitudes, and knowledge were recorded, adolescents’ willingness to consume insect-based food was not significantly increased. Attitudes were identified as the strongest predictor of adolescents’ willingness to consume, while knowledge was not a significant predictor. Conclusions and recommendations that can be applied to other educational interventions are provided to increase the effectiveness of the teaching unit.

## Introduction

Due to the growing world population, it is becoming increasingly urgent to counteract global problems such as climate change, the loss of biodiversity, and hunger (1, 2). All these issues revolve at least partially around food production. To ensure food security for all people, food production must almost double by 2050 (3). As a result, the demand for protein-rich food will also increase (4). Fellows et al. (5) see a need to change the diet of Western societies, which is typically high in meat and dairy products, to one based largely on alternative sustainable proteins. This transition will help mitigate between 10% and 18% of global greenhouse gas emissions attributable to industrial livestock farming (6, 7). In addition to environmental benefits such a change in diet reduces the risk of non-communicable diseases associated with high meat consumption, such as cancer, obesity, and cardiovascular diseases (8–11).

### Insect-based food

Insects – along with cultured meat and dairy products from cellular agriculture, mycoproteins, and plant proteins from many legumes – present such an alternative sustainable protein source to implement a change in diets (12–18). The consumption of edible insects is also called entomophagy.

A direct comparison of the production and consumption of insects and meat shows that insect-based food has many advantages with respect to sustainability indicators, such as lower rates of water and land use (19, 20). More than 2,111 edible insect species are currently known (21, 22); thus information on the sustainability of food from insects cannot be generalized. Data should instead be related to specific insect species (23). For example, yellow mealworms (*Tenebrio molitor*) and crickets (*Acheta domesticus*), which both have a high potential to become established as food in Europe (24), have a higher percentage of edible body mass than conventional livestock such as cattle, pigs, and chickens (25, 26). Furthermore, the production of mealworms and crickets requires less land area and water than the production of meat (27, 28). In addition, insects such as the yellow mealworm can use organic by-products from the agri-food sector as a substrate, which can reduce food waste, competition with other feeds, and overall land use. Using by-products improves the circular economy and promotes sustainability of the food system (29–32). Insect production is equal to conventional livestock farming only in terms of energy input (i.e., the production of mealworms requires a similar amount of energy as the production of meat from pigs or chickens) (26). Insects also provide nutritional benefits over conventional livestock, as their nutrient composition is advantageous. For example, the energy value and protein content of yellow mealworms and crickets are proportionally higher than those of various conventional meat products such as pork shoulder (33). Furthermore, many insect species have high amounts of vitamins and minerals and are rich in essential amino acids, unsaturated fatty acids, and dietary fiber (34–36). These examples show that insect-based food offer great potential for innovation and can contribute to the achievement of the United Nations Sustainable Development Goals (31).

#### Legal framework and willingness to consume

As a result of the adoption of the Novel Food Regulation on January 1, 2018, novel foods based on insects can be approved in the European Union (24). As a current example, in June 2021, the European Food Safety Authority (EFSA) approved dried yellow mealworm larvae (*Tenebrio molitor*) as a novel food (37). Insects are already available on the German market in the form of burger patties, pasta, and protein bars.

The acceptance of insects as food is vital for the introduction of insects as an alternative source of protein in the Western diet. In particular, the focus of research should be on children’s and adolescents’ acceptance of insect-based foods, as they represent the potential future consumers. Several studies have reported varying rates in the willingness to consume insects as food in Western societies (38). Thus far, only a few studies have focused on children’s and adolescents’ willingness to consume insects as food (13, 39, 40). Research has shown that 38.6% of German children and adolescents would be willing to consume an insect burger (40). Although previous studies have demonstrated a higher willingness to consume insect-based food among younger than older people (38), children and adolescents in Germany were less willing to consume an insect burger than adults (40, 41). In line with this, Caparros Megido et al. (39) showed that Belgian adolescents were less willing than adults to eat insects in the future.

#### Nutritional-psychological influencing factors

Several personality traits and nutritional-psychological factors have previously been identified as influencing variables for the willingness to consume insect-based products (38, 42, 43). In the present study, variables were selected that could have an effect on the willingness to consume insects as food. As only a few studies examine the acceptance of insects as food by children and adolescents, some studies with adult participants are also considered below.

Food neophobia describes a person’s aversion to novel and unfamiliar foods, which in most Western cultures includes insects (14, 44, 45). It is generally assumed that food neophobia serves to protect organisms from eating foods perceived to be toxic (46). In many previous studies, food neophobia was found to negatively predict the willingness to consume insect-based food (39–41, 45–48).

The reluctance to use novel and innovative food production methods is known as food technology neophobia (49). In the context of consuming insects, food technology neophobia arises primarily from misconceptions about and fears of modern food technologies as well as a lack of knowledge about the breeding of insects and the production of insect-based food (40, 45). Prior studies have already shown that food technology neophobia appears to be a negative predictor for the willingness to consume insects as food (41, 45, 50, 51).

Food disgust describes a feeling of disgust caused by food-related triggers such as poor hygiene or living contamination (52, 53). In Western countries, insects are often associated with filth and fear of pathogen transmission (54, 55). Previous studies have identified food disgust as an important predictor of the willingness to consume insect-based food among the general population in Western countries (41, 53) and among children and adolescents in Germany (40). However, when simultaneously considering the influence of other nutritional-psychological variables such as food neophobia, food disgust lost its influence on children’s and adolescents’ willingness to consume insect-based food (40).

Attitudes can be defined as a positive or negative affective, cognitive, or conative evaluation of an object, person, group, topic, or idea, in this case insects as food (56). In previous studies, attitudes were identified as a predictor of the willingness to consume insects as food (40, 57, 58). Among children and adolescents, Dupont and Fiebelkorn (40) were able to demonstrate that attitudes toward insects as food and insect-based products are significant factors influencing the willingness to consume insect-based food.

Several studies have already identified the influence of subject matter knowledge about entomophagy on the willingness to consume insect-based food (15, 59–61). Specifically, Kane and Dermiki (59) and Verneau et al. (60) showed that previous knowledge about entomophagy had a positive influence on the willingness to try and the intention to eat insects. In addition, Woolf et al. (61) demonstrated that people aware of the benefits of entomophagy were more willing to consume insect-based food. However, Piha et al. (62) found that knowledge about entomophagy predicts the willingness to buy insects as food among consumers in Northern but not in Central Europe.

#### Information provision *via* educational interventions

Providing information about entomophagy *via* an educational intervention is a common technique to increase the willingness to consume insects as food in Western societies (38). Some previous studies have examined the effect of interventions in non-formal educational or other settings (e.g., tasting or framing studies with product images) on adults’ acceptance of edible insects (48, 57, 60, 63–69). In general, the studies show that adults’ acceptance of edible insects was positively influenced by information on the nutritional and environmental benefits of consuming insects (48, 57, 60, 65, 67). In addition, few studies with adolescents and adults have investigated the extent to which educational interventions influence already proven determinants of the acceptance of insect-based food, such as food disgust (48, 66, 70–74).

Previous studies have shown that food neophobia can be reduced through educational interventions (71, 72). For example, Mustonen et al. (71) investigated the effects of a sensory education intervention over a period of one and a half years on children’s food-related traits and behavior toward (un)familiar food. The intervention consisted of 15 lessons of 90 min each about the importance of the senses for eating and the production of dairy, cereal, and meat. Next discussions occurred as well as practical exercises involving the activation of the senses (71, 75). After the intervention, children’s food neophobia decreased and their willingness to taste unfamiliar food increased.

Cox et al. (73) demonstrated that providing information about novel food technologies for prawn farming *via* simple text descriptions had no effect on adults’ general beliefs about and attitudes toward new food technologies.

Currently, no study examining the effect of an educational intervention on the general food disgust has yet been published. However, Mancini et al. (48) found that young adults’ disgust with eating insects as food was reduced by a 180-min university seminar that included ecological, health, and gastronomic informational content.

Several studies have shown that educational interventions positively influence attitudes toward eating insects (48, 66). For example, in a study specifically investigating the influence of an information intervention on young adults’ attitudes toward eating insects, Mancini et al. (48) found that a 180-min information seminar about ecological, health, and gastronomic aspects of edible insects at a university positively influenced their attitudes.

Previously, several studies found that educational interventions on various environmental and health topics (e.g., marine ecology, water, breast cancer, or organ donation) were effective in increasing participants’ knowledge about these topics (76–79). Woolf et al. (74) found that young adults felt more knowledgeable about entomophagy after an information intervention on the environmental and nutritional advantages as well as the safety aspects of insect consumption (74).

### Aims of the study

Overall, few studies (13, 39, 40) have investigated the influence of (nutritional-)psychological variables on the acceptability of edible insects in children and adolescents. Therefore, a goal of this study is to investigate factors influencing the acceptance of insect-based food by children and adolescents. In particular, this work investigates whether the variables food disgust, food neophobia, food technology neophobia, attitudes, and knowledge influence the willingness to consume insect-based food among children and adolescents. Based on the aforementioned results, it is assumed that food disgust, food neophobia and food technology neophobia have a negative influence. In contrast, knowledge and attitudes are assumed to have a positive influence on the willingness to consume insect-based food.

Furthermore, educational interventions with a focus on providing information could increase the willingness to consume insects as food among children and adolescents (39). However, to the authors’ knowledge, an empirical test of this assumption is not yet available. Therefore, the present study also investigates the influence of a teaching unit in a formal educational setting on the willingness of adolescents to consume insects as food. In addition, the effect on the previously described influencing factors is also examined. The authors hypothesize that food disgust and food neophobia among adolescents will be reduced because of the educational intervention. It is also expected that attitudes will be positively influenced. Furthermore, it is assumed that the knowledge of the adolescents will increase due to the educational intervention. No significant difference is predicted for food technology neophobia.

## Materials and methods

### Sample

A questionnaire-based study in a paper-pencil format was conducted in classes of students from the ninth to the twelfth grade from three secondary schools in Osnabrück (Lower Saxony, Northwest Germany). Two of the schools are in the city of Osnabrück and have more than 1,000 students, while the other school is situated in the district of Osnabrück and has approximately 1,200 students. The study followed a classic pre-, post-, and follow-up test design (see Section “Data collection and study design”). At the pre-test, 142 students participated in the study. Due to incomplete answering of the questionnaire or cases of illness at the post-test and follow-up test, the questionnaires of 28 respondents had to be excluded from the study, resulting in a final sample size of 114 students. The sample is a convenience sample whose size was determined by the availability of the schools in the city and district of Osnabrück and the limited resources of the participating schools (80, 81). The final sample consisted of 58.8% female and 41.2% male respondents with an average age of *M*_*Age*_ = 15.77 years (*SD*_*Age*_ = 1.12 years). Of these subjects, 26 students (22.8%) were flexitarians, six (5.2%) were vegetarians, and two (1.7%) were vegans. All respondents stated that they had heard about eating insects before; thus, the familiarity of the respondents with entomophagy was 100%. A more detailed description of the number of students, grades, age, and gender distribution for each class can be found in Table 1.

**TABLE 1:** Detailed overview of the sample (*N* = 114).

School	Grade	Students	*M*_*Age*_ (*SD*)	Female	Flexitarian	Vegetarian	Vegan	Familiarity^1^
1	9	15	14.14 (0.36)	9	2	1	0	100%
	10	34	15.22 (0.42)	25	12	1	0	100%
	12	10	17.00 (0.67)	6	4	0	0	100%
2	12	31	17.10 (0.67)	16	5	4	2	100%
3	10	24	15.04 (0.36)	11	3	0	0	100%
Total		114	15.77 (1.12)	67	26	6	2	100%

^1^Familiarity with eating insects. The item in the questionnaire was “Have you ever heard that you can eat insects?”.

### Data collection and study design

**FIGURE 1:**
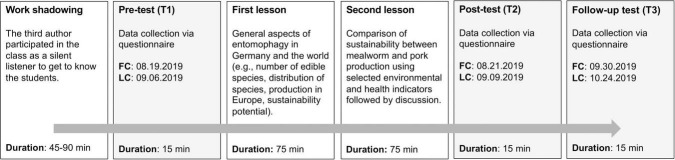
Overview of the study design. FC, first collection of data; LC, last collection of data.

The data collection, including the implementation of the teaching unit with four lessons of 45 min each, took place from August 16 to October 24, 2019. The four lessons were taught as two double lessons of 90 min each. The chronological sequence of the study was divided into six sections: (1) work shadowing, (2) completion of the pre-test (T1), (3) first 90-min lesson, (4) second 90-min lesson, (5) completion of the post-test (T2), and (6) completion of the follow-up test (T3; Figure 1). The average time to complete the questionnaire was 15 min. For school organizational reasons, the completion of the questionnaires had to occur during class hours. Thus, only 75 min remained for each lesson. Consequently, the total duration of the education-only intervention was approximately 150 min.

The post-test was conducted immediately following the teaching unit. The follow-up test was conducted approximately four to six weeks after the post-test survey. Note that due to school holidays and the availability of the respondents, the intervals between the test surveys could not be kept completely consistent. Nevertheless, for all participating classes, the two lessons took place within three to seven days. This procedure made it possible to keep the period of catamnesis (i.e., the measurement period without intervention) quite short. Accordingly, external influences such as reports about the market entry of new insect-based food in magazines, television, or social media were reduced to a minimum.

All classes were taught by the same teacher to exclude possible influences of different teacher personalities or teaching styles. This role was taken by the third author of the study, who is a trained biology teacher. The structure of the teaching unit was standardized by using a teaching script that was adhered to in all participating classes.

The aim of the first lesson was to introduce the terms “entomophagy” and “sustainability” and to discuss the advantages of using insect-based food. For the introduction of the term “entomophagy,” an information sheet on the history of entomophagy and two illustrations about the number and distribution of edible insect species worldwide were used. For the introduction of the term “sustainability,” an information sheet on the nutritional values and sustainability of insects as food was used. With this material, students were asked to explain the term “sustainability” with regard to nutrition and to reflect the advantages and disadvantages of insect-based food. In the second lesson, an explicit comparison between the sustainability potential of mealworms and pork was made. First, students were each shown two pictures of animal-based food and their insect-based equivalents. The students were asked to intuitively rate which food they would prefer. Then, for the explicit comparison, students were given an information sheet on the environmental impact of mealworm and pork production and an illustration of the mealworm production. Furthermore, the production of insect-based food and the disadvantages of traditional meat consumption were discussed. The subsequent discussion was intended to encourage an open dialogue. For example, possible measures to better establish insect-based food in Germany were debated. The teaching structure and the materials used on the topic of “Entomophagy and Sustainability” were based on the teaching concepts of Fiebelkorn and Kuckuck (23, 82) for biology and geography lessons, which have already been published as teaching materials in journals for biology and geography teachers. The original teaching materials, the teaching script, and the questionnaire are provided as Supplementary material.

Permission for data collection and the ethical compliance of the study was confirmed by the responsible state education authority (Lower Saxony State Education Authority; NLSchB) in July 2019 (Reference number: OS 1 R.12–0541/2N). All participating schools were informed about the implementation of the study and written consent was given by the respective headmasters. In addition, written consent was obtained from all students and their parents, and participation in the study was voluntary and consent was revocable at any time. None of the adolescents’ personal data was collected, and students’ anonymity was ensured throughout the study. Pseudonymization of the students was carried out for the assignment of the questionnaires to the three measurement points. The teaching and the questionnaire survey were conducted in either the classroom or the biology lab of the respective class under the supervision of the second and third author and the supervising teachers.

### Questionnaire and variables

The questionnaire was divided into seven sets of questions: (1) introductory explanation on how to fill in the questionnaire and respondents’ personal code for the clear assignment of pre-, post-, and follow-up test; (2) sociodemographic characteristics (age, gender, grade, and school), supplemented with information on diet; (3) nutritional-psychological factors (food disgust, food neophobia, and food technology neophobia); (4) familiarity; (5) students’ willingness to consume; (6) attitudes; and (7) knowledge. The questionnaires had the same order of question blocks for all three measurement points. In the post- and follow-up test questionnaires, the questions about personal nutritional behavior were excluded. Furthermore, the post- and follow-up tests had an open field for additional notes. An overview of the variables collected in the questionnaire and used for the data analysis, including their internal consistency measured as Cronbach’s alpha, is given in Table 2.

**TABLE 2:** Overview and descriptive statistics of all variables (*N* = 114).

Variable	Items	Cronbach’s alpha			Response format
		Pre-test	Post-test	Follow-up test	
Gender	1	–	–	–	”Male” (1) “Female” (2)
Age	1	–	–	–	Open answer format
Familiarity	1	–	–	–	”No, I have never heard of eating insects.” (0) “Yes, I have heard about eating insects.” (1)
Food disgust	8	0.66	0.68	0.69	5-point Likert scale (1 = not disgusting at all, 5 = extremely disgusting)
Food neophobia	10	0.84	0.85	0.87	5-point Likert scale (1 = do not agree at all, 5 = fully agree)
Food technology neophobia	4	0.75	0.81	0.75	5-point Likert scale (1 = do not agree at all, 5 = fully agree)
Attitudes	8 (9)*	0.72 (0.70)*	0.73 (0.75)*	0.74 (0.55)*	5-point Likert scale (1 = do not agree at all, 5 = fully agree)
Knowledge	7	–	–	–	Single choice (tick off)
Willingness to consume	3	0.83	0.85	0.82	5-point Likert scale (1 = very unlikely, 5 = very likely)

*The numbers in parentheses show the number of the original scales’ items and Cronbach’s alphas before elimination due to the principal component analysis.

#### Sociodemographic factors

The sociodemographic data collected were age, gender, school, and grade of the students (Table 2).

#### Familiarity

Familiarity with eating insects was assessed using an item adapted from Verbeke (45): “Have you ever heard that you can eat insects?” The two possible answers were: (1) “No, I have never heard that you can eat insects” and (2) “Yes, I have heard that you can eat insects” (Table 2). Since all students were already familiar with the fact that one can eat insects, the variable was not included in the statistical analysis.

#### Food disgust

In the present study, the German short version of the Food Disgust Scale by Hartmann and Siegrist (53) was used. The adolescents rated their feelings of disgust in each of the following eight “disgust categories” on a five-point Likert scale (Table 2): (1) animal flesh, (2) poor hygiene, (3) human contamination, (4) mold, (5) decaying fruits, (6) fish, (7) decaying vegetables, and (8) living contaminations. For example, “Putting an animal cartilage in your mouth” is the statement in the category “animal flesh.” The scale had an adequate internal reliability, with Cronbach’s alpha values from 0.66 (T1) to 0.69 (T3).

#### Food neophobia

Food neophobia was measured using the ten-item version of the Food Neophobia Test Tool by Damsbo-Svendsen et al. (83), which was explicitly developed for children. On a five-point Likert scale, the adolescents rated to what extent they agreed with statements related to novel foods (Table 2). An example item from the German version of the scale adopted by Dupont and Fiebelkorn (40) is “I like to get to know new and different foods.” Prior to data analysis, all items except “I think unfamiliar food looks unappetizing” and “I am wary of trying food I have not tasted before” were reverse coded. The scale had a high internal consistency, with Cronbach’s alpha values of 0.84 (T1) to 0.87 (T3).

#### Food technology neophobia

Food technology neophobia was assessed using the German short version of the Food Technology Neophobia Scale by Cox and Evans (49), based on Verbeke (45). It consists of four items on a five-point Likert scale (Table 2). An example of an item from this scale is “The benefits of new food technologies are often overstated”. The scale had a high internal consistency, with Cronbach’s alpha values from 0.75 (T1) to 0.81 (T2).

#### Attitudes

The German version (40, 41) of the scale developed by Ruby et al. (58) was used to measure the general attitudes of students toward insect-based food on a five-point Likert scale (Table 2). In total, the scale consists of nine items (e.g., “Eating insects is disgusting”) that can be divided into the following five dimensions: (1) disgust, (2) risks, (3) benefits, (4) morals, and (5) miscellaneous (58). However, following other authors (40, 41, 57), the scale was treated as a unidimensional measure of attitudes toward insect-based food. Based on principal component analysis, the item “Killing insects is unconscionable” was excluded from further analyses due to a component loading of < 0.30 in the follow-up test (84). The elimination of the item led to a considerable increase in Cronbach’s alpha for the modified scale, from 0.55 (T3) to 0.74 (T3). Consequently, this item was also excluded in the analysis of the pre- and post-tests. The Cronbach’s alpha values for the modified attitudes scale in the pre- and post-test were not decreased due to the elimination of the item. The adapted scale showed an acceptable internal consistency, with Cronbach’s alpha values from 0.72 (T1) to 0.74 (T3).

#### Knowledge

Data for measuring students’ knowledge were obtained using a single-choice knowledge test at the end of the questionnaire (Table 2). The test consisted of seven questions with six possible answers each, five distractors and one correct answer. To minimize the error source of guessed answers, it was also possible to select “no idea” as an answer. The questions were created according to the subject matter of the educational intervention and were checked for content, aspiration level, and formulation by several members of the Department of Biology Didactics at Osnabrück University and a biology teacher. The test included four text-based and three number-based tasks. An example of a number task is “Approximately how many different insect species are eaten in the world today?” The respondents could choose between the answer options “500,” “1,000,” “1,500,” “2,000” (correct answer), “2,500,” and “no idea.” Correct answers were coded with “1” and wrong answers and the answer “no idea” were coded with “0”. Finally, a variable was calculated from the sum of all answer codes, which could therefore take values from 0 as the minimum value (all answers wrong) to 7 as the maximum value (all answers correct).

#### Willingness to consume

Based on Lammers et al. (41) and Dupont and Fiebelkorn (40), the willingness to consume was assessed as an aggregated index variable consisting of (1) willingness to try insect-based food, (2) willingness to buy insect-based food, and (3) willingness to substitute meat for food made from insects. Respondents indicated their willingness using a five-point Likert scale (Table 2) with the following questions: “How likely is it that,” (1) “you would try insect-based food?”; (2) “you would buy insect-based food?”; and (3) “you would use insect-based food as a substitute for meat?”. Cronbach’s alpha was α = 0.82 (T3) to α = 0.85 (T2), which indicates a high internal consistency.

### Data analysis

All calculations and analyses were performed using IBM ^®^ SPSS ^®^ Statistics. A significance level of 5% was used for all analyses. Principal component analysis was performed to examine the dimensionality of the scales (84). To examine the normal distribution of all variables for all three measurement points, their respective histograms and Q-Q plots were assessed. This assessment revealed that all variables were approximately normally distributed at the three measurement points. To determine the effectiveness of the teaching unit, a single-factor repeated measures analysis of variance (rmANOVA) was first performed (Table 3). Bonferroni-corrected *post hoc* analyses were used to assess, whether the mean values of the collected variables differed significantly between the three measurement points (T1-T3). In that sphericity could only be assumed for the variables food neophobia and food technology neophobia, a Huynh-Feldt correction of the levels of freedom was carried out for all other variables. In addition, the effect sizes of the pairwise comparisons according to Cohen (85) were calculated. To determine the effect sizes in cases of significant mean differences between the measurement points (T1-T2, T2-T3, T1-T3), paired-samples *t*-tests were performed (84, 86). Moreover, multiple hierarchical regressions were calculated to analyze which potential predictors had an impact on the willingness to consume at each measurement point. The requirements for a multiple regression were all met, including multicollinearity and homoscedasticity. The order of integrating the independent variables in the regression model was based on Dupont and Fiebelkorn (40).

**TABLE 3:** Multiple hierarchical regression explaining the influence of predictors on the willingness to consume at the three points of measurement T1 (pre-test), T2 (post-test), and T3 (follow-up test) (*N* = 114).

WTC-T1	Variables	*B*	*SE B*	β	WTC-T2	Variables	*B*	*SE B*	β	WTC-T3	Variables	*B*	*SE B*	β
Step 1	Constant	4.92***	1.34		Step 1	Constant	7.36***	1.44		Step 1	Constant	6.39***	1.38	
	Age	−0.10	0.08	−0.12		Age	−0.25**	0.09	−0.26		Age	−0.19*	0.08	−0.21
	Gender	−0.44*	0.19	−0.22		Gender	−0.34	0.21	−0.15		Gender	−0.39	0.20	−0.18
Step 2	Constant	6.36***	1.39		Step 2	Constant	8.37***	1.46		Step 2	Constant	7.44***	1.38	
	Age	−0.11	0.08	−0.12		Age	−0.24**	0.09	−0.26		Age	−0.19*	0.08	−0.21
	Gender	−0.26	0.20	−0.13		Gender	−0.19	0.21	−0.09		Gender	−0.24	0.20	−0.12
	FD	−0.47**	0.16	−0.38		FD	−0.43	0.17	−0.24		FD	−0.46**	0.16	−0.27
Step 3	Constant	1.83	1.37		Step 3	Constant	4.32**	1.63		Step 3	Constant	3.29*	1.58	
	Age	−0.02	0.07	−0.03		Age	−0.18*	0.08	−0.19		Age	−0.15*	0.07	−0.17
	Gender	−0.20	0.16	−0.10		Gender	−0.16	0.18	−0.07		Gender	−0.23	0.17	−0.11
	FD	−0.16	0.14	−0.09		FD	−0.08	0.16	−0.04		FD	−0.15	0.16	−0.09
	FN	−0.16	0.13	−0.10		FN	−0.27	0.16	−0.15		FN	−0.14	0.16	−0.08
	FTN	−0.22	0.12	−0.16		FTN	−0.28*	0.12	−0.20		FTN	−0.08	0.12	−0.06
	ATT	0.93***	0.15	0.52		ATT	0.85***	0.20	0.38		ATT	0.88***	0.19	0.46
	KN	−0.10	0.08	−0.10		KN	0.00	0.05	0.00		KN	−0.05	0.06	−0.06
Step 1: *R*^2^_*adj*_. = 0.039; Δ*R*^2^_*adj*_. = 0.039; *p* < 0.05 Step 2: *R*^2^_*adj*_. = 0.101; Δ*R*^2^_*adj*_. = 0.062; *p* < 0.01 Step 3: *R*^2^_*adj*_. = 0.438; Δ*R*^2^_*adj*_. = 0.337; *p* < 0.001					Step 1: *R*^2^_*adj*_. = 0.070; Δ*R*^2^_*adj*_. = 0.070; *p* < 0.01 Step 2: *R*^2^_*adj*_. = 0.115; Δ*R*^2^_*adj*_. = 0.045; *p* < 0.001 Step 3: *R*^2^_*adj*_. = 0.383; Δ*R*^2^_*adj*_. = 0.268; *p* < 0.001					Step 1: *R*^2^_*adj*_. = 0.057; Δ*R*^2^_*adj*_. = 0.057; *p* < 0.05 Step 2: *R*^2^_*adj*_. = 0.121; Δ*R*^2^_*adj*_. = 0.064; *p* < 0.001 Step 3: *R*^2^_*adj*_. = 0.347; Δ*R*^2^_*adj*_. = 0.226; *p* < 0.001				

*p < 0.05, **p < 0.01, ***p < 0.001.

*R*^2^_adj_., adjusted *R*^2^; FD, food disgust; FN, food neophobia; FTN, food technology neophobia; ATT, attitudes; KN, knowledge.

## Results

### Influences of the potential predictors on the willingness to consume

Table 3 summarizes the effects of the potential predictors on the willingness to consume insect-based food. In the pre-test, only one of the seven potential predictors showed a significant influence on the willingness to consume. Thus, attitudes (β = 0.52, *p* < 0.001) could be identified as the strongest predictor for the willingness to consume. In the first step of the multiple regression, 3.9% of the variance of the willingness could be explained by adding the sociodemographic variables age and gender [*F*(2, 106) = 3.20, *p* < 0.05]. By including food disgust in the second step, a further 6.2% of the variance of the willingness to consume could be explained [*F*(3, 105) = 5.04, *p* < 0.01]. An additional 33.7% of the total variance could be explained by adding food neophobia, food technology neophobia, attitudes, and knowledge in the third step [*F*(7, 101) = 13.02, *p* < 0.001]. In total, the overall model for measurement point T1 explained 43.8% of the variance of the willingness to consume.

Regarding the willingness to consume at measurement point T2, three of the seven independent variables could be identified as predictors. Age (β = −0.19, *p* < 0.05) and food technology neophobia (β = −0.20, *p* < 0.05) negatively predicted the willingness to consume, while attitudes (β = 0.38, *p* < 0.001) had a strong positive influence on it. The first step of multiple hierarchical regression clarified 7% of the total variance [*F*(2, 107) = 5.13, *p* < 0.01]. Adding food disgust in the second step could explain an additional 4.5% of the variance [*F*(3, 106) = 5.72, *p* < 0.001]. By including food neophobia, food technology neophobia, attitudes, and knowledge in the third step, an additional 26.8% of the total variance could be explained [*F*(7, 102) = 10.66, *p* < 0.001]. Thus, in total, 38.3% of the variance for adolescents’ willingness to consume at measurement point T2 could be explained with the model.

At measurement point T3, two of the seven potential predictors significantly predicted the willingness to consume. Age negatively predicted the willingness to consume (β = −0.17, *p* < 0.05), while attitudes (β = 0.46, *p* < 0.001) were the strongest predictor for the willingness to consume. The sociodemographic variables in the first step of the analysis could explain 5.7% of the variance [*F*(2, 105) = 4.26, *p* < 0.05]. The nutritional-psychological variable that was included in the second step explained an additional 6.4% of the total variance [*F*(3, 104) = 5.90, *p* < 0.001]. In the third step, a further 22.6% of the variance could be explained [*F*(7, 100) = 9.11, *p* < 0.001] by adding food neophobia, food technology neophobia, attitudes, and knowledge. In total, the model accounted for 34.7% of the variance of the willingness to consume.

### Effectiveness of the teaching unit on “Entomophagy and Sustainability”

Table 4 shows the results of the single factor rmANOVA on the factor time as well as the results of the Bonferroni-corrected *post hoc* analysis. Food disgust significantly changed over the entire measurement period, with a medium effect size [*F*(1.90, 213.07) = 9.62, *p* = ≤ 0.001, *d* = 0.59]. Food disgust meanwhile, had a mean value of 3.14 (*SD* = 0.59) in the pre-test (T1), 3.04 (*SD* = 0.62) in the post-test (T2), and 3.02 (*SD* = 0.64) in the follow-up test (T3). For food neophobia a highly significant change with a large effect size was identified [*F*(2, 226) = 18.32, *p* < 0.001, *d* = 0.81]. Food neophobia had a mean of 2.54 (*SD* = 0.60) at T1, whereas at T2 and T3, the means were 2.40 (*SD* = 0.62) and 2.31 (*SD* = 0.63), respectively. For food technology neophobia, a significant change with a small effect size was observed [*F*(2, 224) = 4.83, *p* < 0.05, *d* = 0.40]. A significant change was only identified between the measurement points T1 and T2 (*MD* = −0.16, *p* < 0.01, *d* = 0.21). The mean value decreased from 2.87 (*SD* = 0.69) to 2.71 (*SD* = 0.79) between T1 and T2. For attitudes toward insect-based food, a highly significant increase with a large effect size was recorded after the educational intervention [*F*(1.68, 188.32) = 52.53, *p* < 0.001, *d* = 1.37]. Examining the changes in attitudes between all measurement points, a highly significant increase was observed between T1 and T2 from 3.52 (*SD* = 0.56) to 3.93 (*SD* = 0.52; *MD* = 0.41, *p* < 0.001, *d* = 0.76). In contrast, a highly significant decrease in the mean scores (*MD* = −0.18, *p* < 0.001, *d* = 0.32) was detected between T2 and T3. The mean values decreased from 3.93 (*SD* = 0.52) to 3.75 (*SD* = 0.55). Knowledge about “Entomophagy and Sustainability” increased highly significantly after the educational intervention, with a large effect size [*F*(1.87, 209.74) = 160.94, *p* < 0.001, *d* = 2.40]. Between T1 and T2, the mean value increased from 1.00 (*SD* = 0.99) to 3.64 (*SD* = 1.71), but at T3 it decreased again to 2.59 (*SD* = 1.46). In the overall analysis, a significant difference with a small effect size was found for the willingness to consume [*F*(1.90, 214.98) = 3.32, *p* < 0.05, *d* = 0.35]. A significant decrease in the willingness to consume was observed between measurement points T2 and T3, with a small effect size (*MD* = −0.14, *p* < 0.05, *d* = 0.12). The mean value decreased from 2.90 (*SD* = 1.11) to 2.77 (*SD* = 1.06) between these two measurement points.

**TABLE 4:** Mean values and standard deviations of the examined variables in T1 (pre-test), T2 (post-test), and T3 (follow-up test) and mean value comparisons with Bonferroni-corrected *post hoc* tests between the individual measurement points (*N* = 114).

Variable	*M* (*SD*)			Main effects		T1-T2			T2-T3			T1-T3		
	T1	T2	T3	*p*	*d*	*MD*	*SE*	*d*	*MD*	*SE*	*d*	*MD*	*SE*	*d*
FD	3.14 (0.59)	3.04 (0.62)	3.02 (0.64)	0.001***	0.59	−0.10***	0.02	0.16	−0.02	0.03	–	−0.12***	0.03	0.19
FN	2.54 (0.60)	2.40 (0.62)	2.31 (0.63)	0.001***	0.81	−0.14***	0.04	0.23	−0.08	0.04	–	−0.22***	0.04	0.36
FTN	2.87 (0.69)	2.71 (0.79)	2.77 (0.76)	0.014*	0.40	−0.16**	0.05	0.21	0.06	0.06	–	−0.10	0.06	–
ATT	3.52 (0.56)	3.93 (0.52)	3.75 (0.55)	0.001***	1.37	0.41***	0.04	0.76	−0.18***	0.03	0.32	0.24***	0.05	0.43
KN	1.00 (0.99)	3.64 (1.71)	2.59 (1.46)	0.001***	2.40	2.64***	0.17	1.86	−1.04***	0.14	0.65	1.59***	0.14	1.25
WTC	2.79 (0.99)	2.90 (1.11)	2.77 (1.06)	0.040*	0.35	0.12	0.06	–	−0.14*	0.05	0.12	−0.02	0.06	–

*p < 0.05, **p < 0.01, ***p < 0.001.

MD, mean value difference; *d*, |*d*|; FD, food disgust; FN, food neophobia; FTN, food technology neophobia; ATT, attitudes; KN, knowledge; WTC, willingness to consume.

## Discussion

### The influence of potential predictors on the willingness to consume

In line with the results of a study by Dupont and Fiebelkorn (40), the gender of the adolescents in this study did not influence their willingness to consume insect-based food. A few studies with adult participants showed that gender only influenced the acceptance of unprocessed insects (41, 87) and specific insect species (88). The present study only asked about the willingness to consume food from insects in general, i.e., no specific product and no specific insect species were predefined. Respondents might have considered different insect-based products and species when answering the question; thus, gender may not have had an influence.

Age negatively predicted the willingness to consume insect-based food at two of the three measurement points (T2 and T3). Previous studies obtained contradictory results regarding the link between participants’ age and their willingness to consume edible insects (39, 40, 48, 89). Whereas Dupont and Fiebelkorn (40) found that older pupils up to the age of 19 were more willing to consume insect-based food, Hartmann et al. (89) reported no influence of age on their adult participants’ willingness to eat insect-based food. However, it has to be considered that many changes in eating behavior particularly occur from ages 10 to 15, when more foods are tried and the food spectrum expands. At those ages the influence of parents on dietary behavior decreases while the influence of peers increases (90–92). Therefore, comparability of our results with findings for adults is limited. In that the span of ages of the adolescents in the present study is only four years, the negative influence of age on adolescents’ willingness to consume insect-based food cannot be generalized.

In contrast to our expectations, food disgust was not a predictor for adolescents’ willingness to consume insect-based food (41, 53). In line with the results of Dupont and Fiebelkorn (40), food disgust influenced adolescents’ willingness to consume only when combined with sociodemographic variables (in the second step of the regression model). Food disgust had no significant influence on the willingness to consume insect-based food after including food (technology) neophobia, attitudes, and knowledge (in the third step of the regression model). According to Dupont and Fiebelkorn (40), this effect could be related to the average age of the participants in this study. Food disgust sensitivity increases with age (52). Therefore, the effect of disgust on willingness to consume could be masked by the effect of other predictors (included in the third step of the regression model). This could explain why food disgust only shows an influence on willingness to consume in the second step of the regression model, but not in the third step.

Several studies have found that food neophobia significantly affects the willingness of adolescents and adults to consume edible insects (40, 41, 47, 48). Surprisingly, in the present study, food neophobia had no significant influence on the willingness of adolescents to consume insect-based food. Similar results were found by Fischer and Steenbekkers (93) and La Barbera et al. (94), who did not find any link between food neophobia and the acceptance of edible insects. In line with Schlup and Brunner (51), we argue that becoming more familiar with the topic of entomophagy might reduce the influence of food neophobia on participants’ willingness to consume edible insects. In the present study, all participants indicated that they were familiar with the topic of entomophagy, so they might not have considered insect-based foods as something new. This view could explain why food neophobia showed no effect on the willingness to consume insect-based food.

Food technology neophobia was identified as a negative predictor for the willingness to consume only at the measurement point directly after the educational intervention. Lammers et al. (41) and Palmieri et al. (50) also identified a negative influence of food technology neophobia on participants’ willingness to consume insect-based food. One possible explanation for our results is that the students were only provided with a small amount of information about the production process for edible insects. Thus, the lack of information and discussion about these novel food technologies might have led to food technology neophobia becoming more influential on the willingness to consume insect-based food. Another explanation could be the limited duration of the educational intervention about two double lessons of 90 min each. Due to this limitation, only selected information on new food technologies could be provided. Thus, the educational intervention should be extended to more than two double lessons in follow-up studies.

As in previous studies (40, 57, 58), attitudes were found to have a positive influence on the willingness to consume insect-based food; indeed, attitudes were the strongest predictor of adolescents’ willingness to consume insect-based food over the entire set of measurements. Based on this result, the promotion of positive attitudes should be focused on. Palmieri et al. (50) suggested that improving knowledge about entomophagy is an effective way to promote positive attitudes toward insects as food. For children and adolescents, this could be achieved by, for example, providing information on entomophagy in formal education. Another way to promote positive attitudes toward insect-based food is holding tasting sessions (40, 50).

In previous studies, knowledge about entomophagy predicted the willingness to consume edible insects (15, 59–61). In contrast, in the present study, adolescents’ knowledge about entomophagy did not influence their willingness to consume insect-based food. However, it should be considered that the mentioned studies assessed “knowledge” about entomophagy in various ways. Kane and Dermiki (59), as well as Verneau et al. (60), examined the influence of participants’ self-reported knowledge of entomophagy on their acceptance. Woolf et al. (61) demonstrated the influence of knowledge about the benefits of entomophagy on the willingness to consume insect-based food. Likewise, Laureati et al. (65) showed that knowledge about the benefits of insect consumption influenced the willingness to consume, although they only observed a minor influence. Since knowledge was collected *via* a knowledge test in this study, the results are not necessarily comparable with those of the other studies. In addition, Piha et al. (62) showed that knowledge about edible insects indirectly influenced the willingness to consume insect-based food through attitudes as a mediator. This supports the assumption that knowledge about entomophagy might positively influence attitudes toward insect-based food but is not sufficient to directly increase participants’ willingness to consume edible insects. Therefore, providing information on entomophagy (e.g., health benefits and production techniques) might be a necessary but not sufficient precondition to influence participants’ willingness to consume edible insects (60, 95). To check this assumption, further analyses such as moderator or mediator analyses would be useful.

### Effectiveness of the teaching unit

The educational intervention affected a number of the variables studied. Specifically, the intervention impacted food disgust, food neophobia, attitudes, and knowledge, with variable effect sizes. While food technology neophobia decreased from pre- to post-test, the willingness to consume was not significantly increased by the educational intervention. In the following section, whether and how the educational intervention was able to elicit changes in the examined variables will be discussed in more detail.

#### Willingness to consume

Contrary to our expectations, adolescents’ willingness to consume insect-based food was not increased through the educational intervention. In contrast, previous studies demonstrated that information interventions about the sustainability potential of insect consumption could positively influence participants’ willingness to consume insect-based products (57, 67). However, for the aforementioned information interventions, it is not clearly identified whether the selection of information, the duration of information provision, the communication about the information, or the interaction of all factors caused the effect on participants’ willingness to consume. Nevertheless, raising awareness of the sustainability potential as well as the environmental and nutritional benefits of insects as food and feed will be crucial in upcoming interventions to influence participants’ willingness to consume insect-based food (53, 65, 67).

In addition to the major role of information about the sustainability, environmental, and nutritional benefits of insect-based food, other factors influencing the willingness to consume insect-based food include participants’ perceived risks, social influence, and lack of familiarity with novel food (technologies) (13, 39, 66, 96). In line with Lensvelt and Steenbekkers (66), we argue that the lack of communication about perceived risks of insect consumption might be the reason that adolescents’ willingness to consume insect-based food could not be increased. Moreover, the social influence of adolescents’ families or peers plays a central role in their dietary habits (13, 97). Thus, adolescents’ willingness to consume insect-based food might be negatively influenced by peers’ rejection of edible insects. For educational interventions, communication about the perceived risks of insect consumption and the production technologies for insect-based food should be included to induce a long-term effect on adolescents’ willingness to consume insect-based food (64, 66, 68). In addition, participants should be enabled to try insect-based food during interventions, as prior consumption of insects has already been identified as an important influencing factor (38).

#### Food disgust

In line with our expectations, adolescents’ food disgust was reduced through the educational intervention in this study. Thus, our results are in line with those of Mancini et al. (48), who found that students’ disgust with eating insects decreased after an informational seminar about the technological, social, and cultural context of insects as food and feed. In contrast to Mancini et al. (48), we demonstrated the novel finding that an educational intervention about entomophagy could affect the more general food-related disgust. Additionally, we found that the decrease in food disgust can last for at least six weeks.

While Rozin and Fallon (98) indicated the decisive role of communication in decreasing the rejection of novel foods, the multifactorial nature of our educational intervention, which consisted of providing information and critical discussion about the production of insect-based food and the disadvantages of traditional meat consumption, might explain the long-term effect on adolescents’ food disgust. In addition to providing information about entomophagy, positive taste experiences should be integrated into educational interventions in the future to enhance long-term decreases in consumers’ food disgust (63). According to Barton et al. (99), insect-based food should be introduced *via* familiar products like chocolate or protein powder to reduce participants’ disgust. At this point, it should be mentioned once again that the Food Disgust Scale (53) measures domain-specific food-related disgust and not disgust with eating insects. Thus, further studies should investigate the effectiveness of the educational intervention in reducing feelings of disgust toward eating insects.

#### Food neophobia

In line with our expectations, adolescents’ food neophobia could be reduced by educational intervention. Mustonen et al. (71) and Park and Cho (72) also found that sensory and taste education programs with repetitive treatments such as private discussions could reduce children’s food neophobia. However, which component(s) of the educational intervention in the present and the aforementioned studies caused the effect is not clear. Nevertheless, combining information supply, discussions, and practical exercises seems to be critical for reducing food neophobia. This assumption is in line with the results by Arena et al. (70), who demonstrated that the mere provision of ecological and nutritional information about edible insects – without critical discussions and exercises – did not influence adults’ food neophobia.

Furthermore, Verbeke (45) indicated that exposure to insects as a novel food in educational interventions could be useful to reduce food neophobia. Therefore, incorporating sensory and tasting experiences with tasty insect-based products in educational interventions could potentially reduce food neophobia even more than observed in the present study.

#### Food technology neophobia

In contrast to our expectations, food technology neophobia was reduced after the educational intervention, although the effect size was small. Notably, our expectations were based on the study by Cox et al. (73), in which providing additional information about novel food technologies in a different context (prawn farming) had no effect on participants’ general beliefs about and attitudes toward novel food technologies.

According to the House of Lords (100), fear of novel food technologies arises in people essentially due to a lack of information about those very technologies. Thus, the small decrease in adolescents’ food technology neophobia between the pre- and post-test might be explained by the provided information and discussion about the cultivation and processing of mealworms for food production. Thus, to reduce children’s and adolescents’ food technology neophobia effectively, educational interventions should provide information on novel food technologies. According to Vidigal et al. (96), this information should include descriptions of the new technologies as well as their advantages and disadvantages. Moreover, the positive environmental impact should be emphasized to reduce existing fears associated with novel food technologies (73). In addition, according to Siegrist (101), educational interventions should elicit confidence in novel technologies since confidence is the key factor in the acceptance of new food technologies.

#### Attitudes

In line with our expectations, adolescents’ attitudes toward insect-based food could be improved by educational intervention. Mancini et al. (48) also found that attitudes toward insects as food increased through an information intervention. In contrast to the information intervention by Mancini et al. (48) about ecological, health, and gastronomic aspects of edible insects, in the present study, the educational intervention consisted of information and discussion about the sustainability potential of eating insects in comparison to eating traditional meat. The educational intervention in the present study comprised several treatments (information, social interaction, and discussion), which could explain the large positive effect on adolescents’ attitudes (102). Moreover, students’ familiarity with entomophagy before the educational intervention might have enhanced the large positive change in attitudes toward insect-based food (63).

In addition, previous studies by Barsics et al. (63) and Lensvelt and Steenbekkers (66) demonstrated that participants’ attitudes toward entomophagy could be positively influenced by tasting sessions with insect-based products coupled with information sessions about entomophagy. In contrast, information-only sessions about edible insects had no influence. Consequently, educational interventions should not consist of only an information session about entomophagy, as multifactorial treatments, especially those including tasting sessions, are more effective in improving participants’ attitudes toward insect-based food (13, 39, 63, 66). Moreover, to foster positive attitudes toward insect-based food *via* educational interventions, it is vital to provide adolescents with knowledge about and awareness of entomophagy (50, 95).

#### Knowledge

In line with our expectations and the results of previous studies, adolescents’ knowledge about entomophagy was increased following the educational intervention (76–79). In previous studies, various methods, including presentations, discussions, and outdoor activities, were utilized (61, 76–79). According to this, the variety of methods and tools used in the present study, such as worksheets with information on eating insects and discussions about the sustainability potential of edible insects, could be a possible explanation for the significant increase in adolescents’ knowledge. However, it should be noted that the knowledge test was the same at all three measurement points. Thus, regardless of the quality and content of the information, it could be assumed that the respondents would achieve a better result if they repeatedly answered the same questions in the knowledge test in the post- and follow-up tests. Although students’ knowledge levels flattened slightly at the follow-up test, it was possible to increase adolescents’ knowledge on entomophagy in the long term.

### Limitations

There are some limitations regarding the representativeness of the data and the methodology of the study. First, it must be mentioned that only students at secondary schools from the ninth to the twelfth grades were examined. Therefore, the results are not representative of students at other types of schools or in lower grades. Additionally, since the study design is decidedly labor-intensive, the sample was limited to three secondary schools from Osnabrück. Therefore, the final sample size of 114 students limits the power and representativeness of the results. However, the major limitation of the present study is the lack of a control group due to the study design. Therefore, it is not possible to compare the results we obtained with those from a group of students who did not participate in the educational intervention during the survey period. Hence, changes might not be due exclusively to the educational intervention; rather, other factors such as group effects may have played a role. Notably, however, obtaining a control group for an intervention in a formal educational setting proves to be difficult. Consequently, school-based educational intervention studies are often conducted without a control group (103–105). For follow-up studies examining the effect of this educational intervention, larger sample size, and inclusion of a control group in the study design are recommended to increase the representativeness and power of the results.

A further limitation is related to the long-term effect of the educational intervention. Due to the period of only six weeks between the post- and follow-up tests, it is only possible to speak of a long-term effect to a limited extent. Another limitation is that it cannot be determined whether the information or the discussion about the sustainability potential of eating insects in comparison to eating traditional meat is responsible for the effect of the intervention. Müller (102) already showed by analyzing intervention studies in environmental psychology that multifactorial treatments consisting of information supply, personal interaction, and feedback can be more effective than single factorial treatments. Previous intervention studies showed a positive effect on participants’ acceptance of edible insects by providing information on the nutritional and environmental benefits of insect consumption (57, 67). Therefore, future intervention studies should investigate whether a multifactorial treatment is more effective in promoting participants’ acceptance of edible insects than a single factorial treatment. In addition, it should be noted that repeated exposure to the topic of insects as food during the multifactorial treatments might also have enhanced adolescents’ preference and positive attitudes toward insects as food (106). Therefore, this study cannot clearly differentiate whether the effect was due to the multifactorial treatments or the repeated exposure with the topic “edible insects.”

As another limitation it should be mentioned that the reliability values of the Food Disgust Scale applied in this study were lower compared to previous studies with adults (41, 53, 107). The reliability values are in line with the values from Dupont and Fiebelkorn (40), who also investigated adolescents. Therefore, the Food Disgust Scale was included in the analysis. Nevertheless, it should be considered that the Food Disgust Scale by Hartmann and Siegrist (53) was developed for adults. The low reliability values might indicate that the scale is not suitable for adolescents. For upcoming studies, other scales measuring food disgust in children and adolescents, such as those by Muris et al. (108) and Viar-Paxton et al. (109), should be used.

## Conclusion

Despite the limitations mentioned above, the study makes an important contribution to research on the effect of educational interventions and their impact on adolescents’ attitudes and willingness to consume insect-based food. To our knowledge, the present study is the first study on this topic to be conducted in the formal education sector in Germany. The study was able to demonstrate that a relatively short teaching unit of 150 min is sufficient to influence students’ food disgust, food neophobia, food technology neophobia, and attitudes toward and knowledge about insect-based food. Specifically, the results showed that adolescents’ domain-specific food disgust and food neophobia significantly decreased over time through the educational intervention. In addition, adolescents’ food technology neophobia could be significantly reduced in the short term through the educational intervention. Moreover, adolescents’ attitudes toward and knowledge about edible insects significantly increased through the educational intervention, with the largest effect observed over time. In contrast, the willingness of adolescents to consume insect-based food only increased immediately after the educational intervention. Regarding adolescents’ willingness to consume, attitudes toward insect-based food were the most important predictor, while age and food technology neophobia were also identified as influential factors. Despite our expectations, adolescents’ food disgust and food neophobia had no influence on the willingness to consume insect-based food after inclusion of the nutritional-psychological variables. Based on the results, educational interventions regarding entomophagy should not focus solely on promoting knowledge about insect consumption, as this had no direct effect on the willingness to consume insect-based food (15, 60, 61). Thus, educational interventions should focus not only on providing information, but also on promoting positive attitudes toward insects as an alternative protein source. This goal could be achieved by including tasting sessions in educational interventions, as previous studies have shown that tasting edible insects and insect-based products had a positive impact on attitudes toward insects as food (39, 63, 66). Why adolescents’ willingness to consume insect-based food did not increase over time through the educational intervention cannot be explained, however, even though attitudes toward insect-based food significantly increased.

## Data availability statement

The raw data supporting the conclusions of this article will be made available by the authors, without undue reservation. The Supplementary material translated into English can be obtained from the corresponding author upon request.

## Ethics statement

The studies involving human participants were reviewed and approved by Lower Saxony State Education Authority (NLSchB). Written informed consent to participate in this study was provided by the participants’ legal guardian/next of kin.

## Author contributions

LS: formal analysis, writing – original draft preparation, and writing – review and editing. JD: data curation and writing – review and editing. FS: conceptualization, investigation, and writing – original draft preparation. HH: investigation and writing – original draft preparation. MB: formal analysis and writing – review and editing. FF: conceptualization, writing – review and editing, project administration, resources, and supervision. All authors contributed to the article and approved the submitted version.
