# Supplementary material for: Effectiveness of a teaching unit on the willingness to consume insect-based food – An intervention study with adolescents from Germany
Source: Front Nutr. 2022 Oct 5;9:889805. doi: 10.3389/fnut.2022.889805 (PMC9583909; doi:10.3389/fnut.2022.889805)
Supplement: Supplementary file 1 [file Data_Sheet_1.PDF]

1669288280

Neuartige Lebensmittel aus Insekten

Deine Meinung ist gefragt!

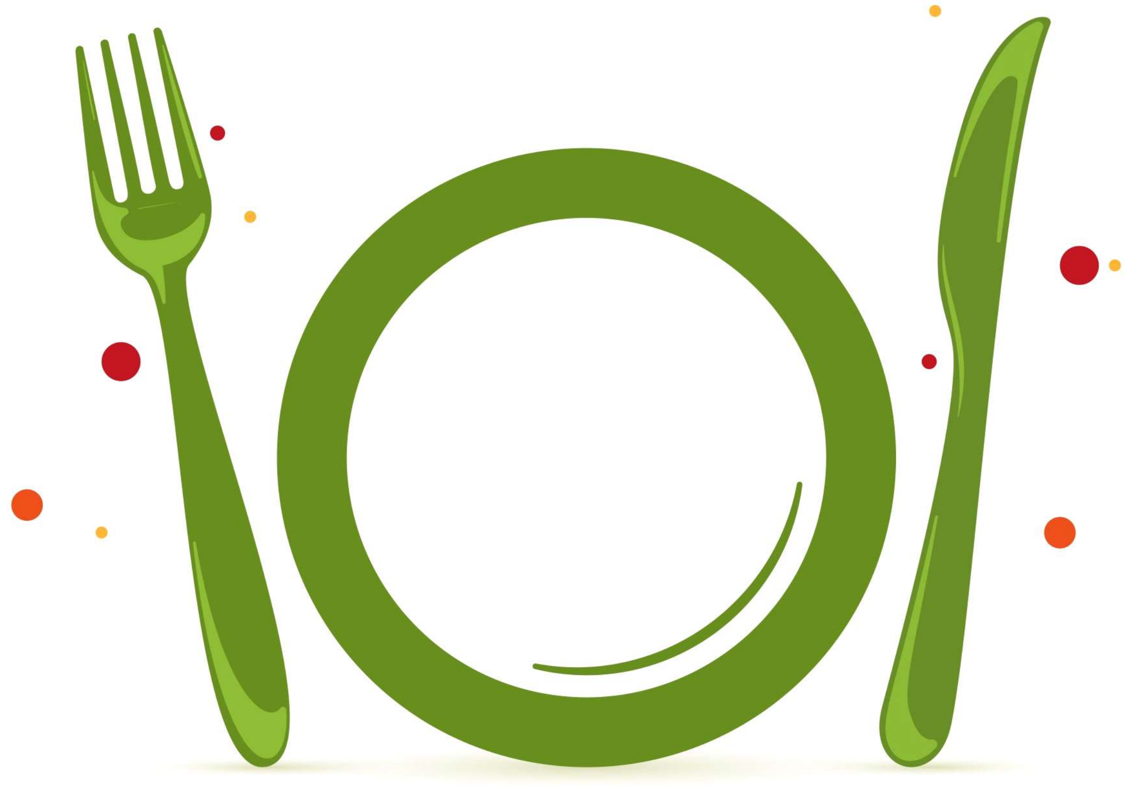

Der gleiche Fragebogen noch einmal?

Liebe Teilnehmerin, lieber Teilnehmer,

Ich hoffe, dass dir die Unterrichtseinheit Spaß gemacht hat und du ein bisschen mitnehmen konntest. Auch wenn du den Fragebogen schon vor der Unterrichtseinheit ausgefüllt hast, würde ich dich darum bitten, den Fragebogen noch einmal genauso ernsthaftauszufüllen wie am Anfang. Für meine Studie brauche ich den Vergleich der beiden Fragebögen, um zu untersuchen, ob der Unterricht Auswirkungen auf die Einstellungen gegenüber Nahrungsmitteln aus Insekten hatte.

Wenn du im Anschluss noch Fragen hast, kontaktiere mich gerne.

E-Mail: feschade@uos.de

Vielen Dank für deine Mithilfe!

Hier ist wieder dein **persönlicher Code** gefragt, damit ich die zwei Fragebögen vergleichen kann:

Die ersten zwei Buchstaben des Namens deiner Mutter ☐☐

Dein Geburtstag (nur der Tag, z.B. 05.August = 05) ☐☐

Die letzten Buchstaben des Namens deines Vaters ☐☐

0226288288

1. Ein paar Fragen für die Statistik

Ich gehe in die: ________ **Klasse.**

Meine Schule heißt: ____________________

Beabsichtigst du deinen Fleischkonsum (Rind, Schwein, Geflügel) in nächster Zeit zu reduzieren?

☐ Ja ☐ Nein ☐ Ich esse sowieso kein Fleisch, weil ich Vegetarier/Veganer bin.

Hast du im Rahmen dieser Unterrichtseinheit Insekten probiert?

☐ Ja ☐ Nein

Wenn ja, wann?

☐ am Anfang ☐ am Ende

2. Wie schätzt du dein Umweltverhalten ein?

	Stimme überhaupt nicht zu	Stimme eher nicht zu	teils/ teils	Stimme eher zu	Stimme voll zu
Es ist mir wichtig, dass die von mir verwendeten Produkte die Umwelt nicht belasten.	☐	☐	☐	☐	☐
Ich berücksichtige die potenziellen Umweltauswirkungen meines Handelns bei vielen meiner Entscheidungen.	☐	☐	☐	☐	☐
Meine Kaufgewohnheiten werden durch meine Sorge um die Umwelt beeinflusst.	☐	☐	☐	☐	☐
Ich bin besorgt darüber, die Ressourcen unseres Planeten zu verschwenden.	☐	☐	☐	☐	☐
Ich würde mich selbst als umweltbewusst bezeichnen.	☐	☐	☐	☐	☐
Ich bin bereit, Unannehmlichkeiten in Kauf zu nehmen, um umweltfreundliche Entscheidungen zu treffen.	☐	☐	☐	☐	☐

3. Wie ekelig findest du die folgenden Produkte und Situationen?

	überhaupt nicht ekelig	nicht ekelig	weder noch	ekelig	extrem ekelig
Apfelstücke, die sich an der Luft verfärbt haben.	☐	☐	☐	☐	☐
Die Vorstellung mit unsauberem Besteck in einem Restaurant zu essen.	☐	☐	☐	☐	☐
Das Essen, welches mir ein Nachbar geschenkt hat, den ich kaum kenne.	☐	☐	☐	☐	☐
Käse essen, von welchem zuvor Schimmel weggeschnitten wurde.	☐	☐	☐	☐	☐
Einen Tierknorpel in den Mund nehmen.	☐	☐	☐	☐	☐
Die Konsistenz einiger Fischarten im Mund.	☐	☐	☐	☐	☐
Braunverfärbtes Fruchtfleisch von einer Avocado essen.	☐	☐	☐	☐	☐
Eine kleine Schnecke in meinem Salat, den ich gerade esse.	☐	☐	☐	☐	☐

4727288288

4. Wie reagierst du auf neuartige Lebensmittel?

	Stimme überhaupt nicht zu	Stimme eher nicht zu	teils/ teils	Stimme eher zu	Stimme voll zu
Ich probiere gerne Lebensmittel aus, die ich noch nie zuvor gegessen habe.	☐	☐	☐	☐	☐
Ich mag es, neue und verschiedene Lebensmittel kennenzulernen.	☐	☐	☐	☐	☐
Ich denke, es macht Spaß, Lebensmittel auszuprobieren, die ich nicht kenne.	☐	☐	☐	☐	☐
Ich probiere oft Lebensmittel, auch wenn ich nicht weiß, was es ist.	☐	☐	☐	☐	☐
Ich habe keine Angst davor, Dinge zu essen, die ich noch nie zuvor gegessen habe oder nicht kenne.	☐	☐	☐	☐	☐
Es macht mir nichts aus, Lebensmittel zu essen, die ich nicht kenne.	☐	☐	☐	☐	☐
Ich esse viele verschiedene Lebensmittel.	☐	☐	☐	☐	☐
Ich fürchte mich davor, Speisen zu essen, die ich vorher noch nie gegessen habe.	☐	☐	☐	☐	☐
Ich denke, unbekanntes Essen sieht oft unappetitlich aus.	☐	☐	☐	☐	☐
Ich habe keine Angst davor, Lebensmittel zu probieren, die ich noch nie probiert habe.	☐	☐	☐	☐	☐

5. Was denkst du über Technologien zur Produktion von neuartigen Nahrungsmitteln?

	Stimme überhaupt nicht zu	Stimme eher nicht zu	teils/ teils	Stimme eher zu	Stimme voll zu
Es gibt bereits viele schmackhafte Lebensmittel, so dass wir keine neuen Lebensmitteltechnologien brauchen, um mehr zu produzieren.	☐	☐	☐	☐	☐
Die Vorteile von neuen Lebensmitteltechnologien werden häufig übertrieben dargestellt.	☐	☐	☐	☐	☐
Neue Lebensmitteltechnologien mindern die natürliche Qualität von Lebensmitteln.	☐	☐	☐	☐	☐
Es macht keinen Sinn, Hightech-Lebensmittel auszuprobieren, weil die, die ich esse, bereits gut genug sind.	☐	☐	☐	☐	☐

1296288286

6. Wären Insekten als Nahrungsmittel etwas für dich und deine Eltern?

**Wie wahrscheinlich ist es, dass...**	sehr unwahrscheinlich -2	-1	0	1	sehr wahrscheinlich 2
...du Nahrungsmittel aus Insekten **probieren** würdest?	☐	☐	☐	☐	☐
...du Nahrungsmittel aus Insekten als **kaufen** würdest?	☐	☐	☐	☐	☐
...du Nahrungsmittel aus Insekten als **Fleischersatz** nutzen würdest?	☐	☐	☐	☐	☐

Wie einfach wäre es für dich, Nahrungsmittel aus Insekten in deine gewohnte Ernährungsweise einzubauen?

sehr schwierig	schwierig	eher schwierig	eher einfach	einfach	sehr einfach
☐	☐	☐	☐	☐	☐

In den nächsten drei Fragen wird nach deiner Einschätzung zum Verhalten deiner Eltern gefragt.

**Wie wahrscheinlich ist es, dass...**	sehr unwahrscheinlich -2	-1	0	1	sehr wahrscheinlich 2
...deine Eltern Nahrungsmittel aus Insekten **probieren** würden?	☐	☐	☐	☐	☐
... deine Eltern Nahrungsmittel aus Insekten im Alltag **kaufen** würden?	☐	☐	☐	☐	☐
...deine Eltern Nahrungsmittel aus Insekten als **Fleischersatz** nutzen würden?	☐	☐	☐	☐	☐

Was meinst du, wie einfach wäre es für deine Eltern, Nahrungsmittel aus Insekten in ihre gewohnte Ernährungsweise einzubauen?

sehr schwierig	schwierig	eher schwierig	eher einfach	einfach	sehr einfach
☐	☐	☐	☐	☐	☐

6775288283

7. Wie ist deine persönliche Einstellung zu Nahrungsmitteln aus Insekten?

Im Folgenden werden dir zu deiner persönlichen Einstellung gegenüber Nahrungsmitteln aus Insekten immer zwei gegensätzliche Wortpaare gezeigt, wie z.B. "ekelig-lecker". In diesem Beispiel müsstest du dich also entscheiden, ob du Nahrungsmittel aus Insekten "eher ekelig" oder "eher lecker" findest.

Wie ist deine persönliche Einstellung gegenüber Nahrungsmitteln aus Insekten?

Nahrungsmittel aus Insekten sind / haben...

	−					+		
	-3	-2	-1	0	1	2	3	
ekelig	☐	☐	☐	☐	☐	☐	☐	lecker
ungesund	☐	☐	☐	☐	☐	☐	☐	gesund
unhygienisch	☐	☐	☐	☐	☐	☐	☐	hygienisch
primitiv	☐	☐	☐	☐	☐	☐	☐	zivilisiert
geringen Nährwert	☐	☐	☐	☐	☐	☐	☐	hohen Nährwert
nicht nachhaltig	☐	☐	☐	☐	☐	☐	☐	nachhaltig
uninteressant	☐	☐	☐	☐	☐	☐	☐	interessant
keine Zukunft	☐	☐	☐	☐	☐	☐	☐	Zukunft
altmodisch	☐	☐	☐	☐	☐	☐	☐	modern
schlecht für andere Tiere	☐	☐	☐	☐	☐	☐	☐	gut für andere Tiere

Und jetzt noch ein paar letzte Fragen zu deinen Einstellungen...

	Stimme überhaupt nicht zu	Stimme eher nicht zu	teils/ teils	Stimme eher zu	Stimme voll zu
Insekten zu essen ist ekelhaft.	☐	☐	☐	☐	☐
Der Verzehr von Insekten erhöht das Risiko einer Krankheit, die durch Erreger hervorgerufen wird.	☐	☐	☐	☐	☐
Insekten enthalten schädliche Krankheitserreger.	☐	☐	☐	☐	☐
Insekten enthalten Schadstoffe.	☐	☐	☐	☐	☐
Insekten haben sehr viele Nährstoffe.	☐	☐	☐	☐	☐
Insekten zu essen ist gut für die Umwelt.	☐	☐	☐	☐	☐
Insekten zu töten ist gewissenlos.	☐	☐	☐	☐	☐
Insekten sind in der Lage Schmerzen zu spüren.	☐	☐	☐	☐	☐
Es ist für den Menschen nicht natürlich, Insekten zu essen.	☐	☐	☐	☐	☐

6581288288

8. Das Mini-Entomophagie-Quiz

Im Folgenden findest du ein paar Fragen zu Insekten als Nahrungsmittel. Dabei möchte ich nur ermitteln, wie viel du durch meinen Unterricht dazugelernt hast, du sollst keineswegs bloßgestellt werden. Am Ende meiner Unterrichtseinheit werde ich die gleichen Fragen nochmal stellen. Es gibt immer fünf Antwortmöglichkeiten, von der genau **eine richtig** ist. Wenn du die Frage nicht beantworten kannst, rate bitte nicht, sondern kreuze "keine Ahnung" an.

(1) Wie viele verschiedene Insektenarten werden heutzutage ca. auf der Welt gegessen?

- ☐ 500
- ☐ 2000
- ☐ 1000
- ☐ 2500
- ☐ 1500
- ☐ Keine Ahnung

(2) Welches Tier hat prozentual den höchsten essbaren Anteil?

- ☐ Mehlwurm
- ☐ Schwein
- ☐ Heimchen
- ☐ Rind
- ☐ Huhn
- ☐ Keine Ahnung

(3) Seit wann können Insekten als Nahrungsmittel in Deutschland zugelassen werden?

- ☐ 2015
- ☐ 2018
- ☐ 2016
- ☐ 2019
- ☐ 2017
- ☐ Keine Ahnung

(4) Welche Aussage ist falsch?

- ☐ Industrieländer haben 2030 einen Fleischkonsum von 100kg/ Kopf/ Jahr.
- ☐ Industrieländer haben einen doppelt so hohen Fleischkonsum wie der Durchschnitt der Welt.
- ☐ Schwellenländer essen zunehmend mehr Fleisch.
- ☐ Der Fleischkonsum von Entwicklungsländern steigt nicht an.
- ☐ Der Fleischkonsum von Schwellenländern liegt oberhalb des Weltdurchschnitts.
- ☐ Keine Ahnung

(5) Wie viele Tiere werden in Deutschland täglich geschlachtet?

- ☐ 100.000
- ☐ 2.000.000
- ☐ 500.000
- ☐ 2.500.000
- ☐ 1.000.000
- ☐ Keine Ahnung

(6) Welche Gruppe von Insekten wird auf der Welt am häufigsten gegessen?

- ☐ Schaben
- ☐ Käfer
- ☐ Heuschrecken
- ☐ Termiten
- ☐ Libellen
- ☐ Keine Ahnung

(7) Welche Aussage zu einer "Nachhaltigen Entwicklung" ist falsch?

- ☐ Nachhaltiges Handeln betrifft die Bedürfnisse heutiger und zukünftiger Generationen.
- ☐ An dem Aktionsprogramm zur Nachhaltigen Entwicklung sind über 150 Staaten beteiligt.
- ☐ Bei einer Nachhaltigen Entwicklung geht es hauptsächlich darum, Wirtschaft und Umweltschutz zu verbinden.
- ☐ Nachhaltige Entwicklung ist ein Handlungsprinzip zur Ressourcennutzung.
- ☐ Es ist nachhaltig, wenn jeder Mensch die gleichen Chancen bekommt.
- ☐ Keine Ahnung

Wenn du noch etwas zur Umfrage oder zum Thema sagen möchtest, ist hier Platz dafür:

Vielen Dank!

<< < Zurücksetzen Speichern unter > >>

6045288283
