# Supplementary material for: Effectiveness of a teaching unit on the willingness to consume insect-based food – An intervention study with adolescents from Germany
Source: Front Nutr. 2022 Oct 5;9:889805. doi: 10.3389/fnut.2022.889805 (PMC9583909; doi:10.3389/fnut.2022.889805)
Supplement: Supplementary file 3 [file Data_Sheet_3.PDF]

Maikäfersuppen, ein vortreffliches und kräftiges Nahrungsmittel

Man sollte nicht glauben, dass der gemeine Maikäfer (*Melolontha vulgaris Fabr. Scarabaeus Melolontha Linn.*), welcher oft eine verderbliche Landplage ist, und Alles verheert, eine so gute Suppe liefern könnte, wie solche wirklich von ihm gewonnen, hier von Vielen bereitet und mit Vergnügen gegessen wird.
Unsere Studenten essen Maikäfer nach abgerissenen Füssen roh, ganz wie sie sind, und nicht wenige ohne den geringsten Nachtheil; in vielen Conditoreien sind sie überzuckert zu haben, und man isst sie candirt an Tafeln zum Nachtische.
Die Maikäfersuppe wird bereitet, wie jene der Krebse. Die Käfer, von welchen man 30 Stück auf eine Person rechnet, werden, so wie sie gefangen sind, gewaschen, dann ganz in einem Mörser gestoßen, in heißer Butter hart geröstet und in Fleischbrühe aufgekocht, fein durchgeseiht und über gerostete Semmelabschnitte angerichtet. Ist die Bouillon auch schlecht, so wird sie doch durch die Kraft der Maikäfer vorzüglich, und eine Maikäfersuppe, gut bereitet, ist schmackhafter, besser und kräftiger, als eine Krebssuppe; ihr Geruch ist angenehm, ihre Farbe ist bräunlich, wie die der Maikäferflügel.
Nur Vorurtheil konnte dieses feine und treffliche Nahrungsmittel, namentlich für sehr entkräftete Kranke, diesen entziehen, und ist das Vorurtheil dagegen einmal besiegt, so wäre diese Suppe eine gute Acquisition für Hospitäler und Kasernen, wo sie, auch ohne Bouillon, mit Wasser bereitet, herrliche Dienste thun wird, und ich sehe gar nicht ein, warum man die Maikäfer so verachtet hat und noch verachtet. Sehen sie ekelhafter aus, als die Schildkröten, aus welchen die so berühmten und theuren Kraftsuppen bereitet werden? Alle Gäste, welche bei mir, ohne es zu wissen und ohne es zu erfahren, Maikäfersuppen genossen haben, verlangten doppelte, ja dreifache Portionen!

Dr. Schneider

Lena Szczepanski – Osnabrück University – Department of Biology Didactics
Email: lena.szczepanski@uni-osnabrueck.de

Täglich konsumieren weltweit bis zu zwei Milliarden Menschen in über 130 Ländern Insekten als Teil ihrer traditionellen Ernährung, vor allem in Asien und Afrika sowie in Süd- und Mittelamerika. Mexiko gilt mit mehr als 549 zum Verzehr genutzter Insektenarten als weltweiter Spitzenreiter der Entomophagie (griech. *entomos* = Insekt, *phagein* = essen) (Material 2).

Schätzungen zufolge werden derzeit insgesamt 2.111 verschiedene Insektenarten als Nahrungsmittel genutzt. Am häufigsten werden Käfer, Schmetterlinge, Ameisen, Bienen und Wespen gegessen. Von den eben genannten Insekten-Gruppen werden oftmals die Larven- und Puppenstadien verspeist. Von Heuschrecken, der am vierthäufigsten konsumierten Insekten-Gruppe, werden meistens die ausgewachsenen Tiere gegessen (Material 3).

Was für viele Menschen auf der Welt als völlig normal angesehen wird, stößt in vielen Ländern Europas, inklusive Deutschland, noch auf Ablehnung. Dabei lehrt uns die Geschichte etwas anderes: Selbst in Deutschland wurden Insekten noch bis Mitte des 20. Jahrhunderts als Nahrungsmittel genutzt, zum Beispiel waren Maikäfer eine beliebte Speise bei Studierenden, egal, ob lebendig, mit Zucker kandiert oder als Suppe (Material 1). Aus historischer Sicht ist es daher eher überraschend, dass in Europa und Deutschland kaum Insekten konsumiert werden. In Deutschland wurde mit der seit dem 1. Januar 2018 in Kraft getretenen, europaweit geltenden „*Novel-Food*-Verordnung“ der erste Schritt in Richtung einer „Wiedereingliederung“ von Insekten als Nahrungsmittel getan. Fortan können Nahrungsmittel aus Insekten auch in Deutschland zugelassen werden. Erste große Supermarktketten, wie z.B. REWE, haben die Chance genutzt und bieten seit Beginn des Jahres erste Nahrungsmittel aus Insekten an (z. B. Insektenburger und Mehlwurm-Pasta). Es ist anzunehmen, dass weitere Supermärkte nachziehen und sich die Produktpalette in den kommenden Jahren weiter vergrößern wird.

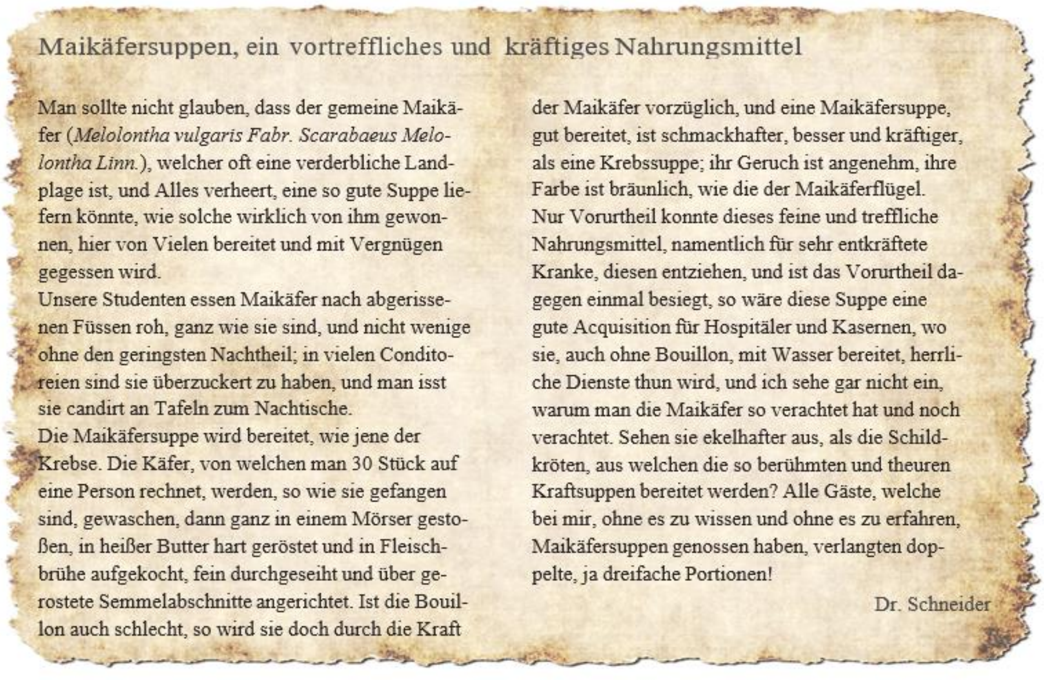

Maikäfersuppen, ein vortreffliches und kräftiges Nahrungsmittel

Man sollte nicht glauben, dass der gemeine Maikäfer (*Melolontha vulgaris Fabr. Scarabaeus Melolontha Linn.*), welcher oft eine verderbliche Landplage ist, und Alles verheert, eine so gute Suppe liefern könnte, wie solche wirklich von ihm gewonnen, hier von Vielen bereitet und mit Vergnügen gegessen wird.
Unsere Studenten essen Maikäfer nach abgerissenen Füssen roh, ganz wie sie sind, und nicht wenige ohne den geringsten Nachtheil; in vielen Conditoreien sind sie überzuckert zu haben, und man isst sie candirt an Tafeln zum Nachtische.
Die Maikäfersuppe wird bereitet, wie jene der Krebse. Die Käfer, von welchen man 30 Stück auf eine Person rechnet, werden, so wie sie gefangen sind, gewaschen, dann ganz in einem Mörser gestoßen, in heißer Butter hart geröstet und in Fleischbrühe aufgekocht, fein durchgeseiht und über geröstete Semmelabschnitte angerichtet. Ist die Bouillon auch schlecht, so wird sie doch durch die Kraft der Maikäfer vorzüglich, und eine Maikäfersuppe, gut bereitet, ist schmackhafter, besser und kräftiger, als eine Krebssuppe; ihr Geruch ist angenehm, ihre Farbe ist bräunlich, wie die der Maikäferflügel.
Nur Vorurtheil konnte dieses feine und treffliche Nahrungsmittel, namentlich für sehr entkräftete Kranke, diesen entziehen, und ist das Vorurtheil dagegen einmal besiegt, so wäre diese Suppe eine gute Acquisition für Hospitäler und Kasernen, wo sie, auch ohne Bouillon, mit Wasser bereitet, herrliche Dienste thun wird, und ich sehe gar nicht ein, warum man die Maikäfer so verachtet hat und noch verachtet. Sehen sie ekelhafter aus, als die Schildkröten, aus welchen die so berühmten und theuren Kraftsuppen bereitet werden? Alle Gäste, welche bei mir, ohne es zu wissen und ohne es zu erfahren, Maikäfersuppen genossen haben, verlangten doppelte, ja dreifache Portionen!

Dr. Schneider

Material 1: Rezept für eine Maikäfersuppe aus dem Jahr 1844

Lena Szczepanski – Osnabrück University – Department of Biology Didactics
Email: lena.szczepanski@uni-osnabrueck.de

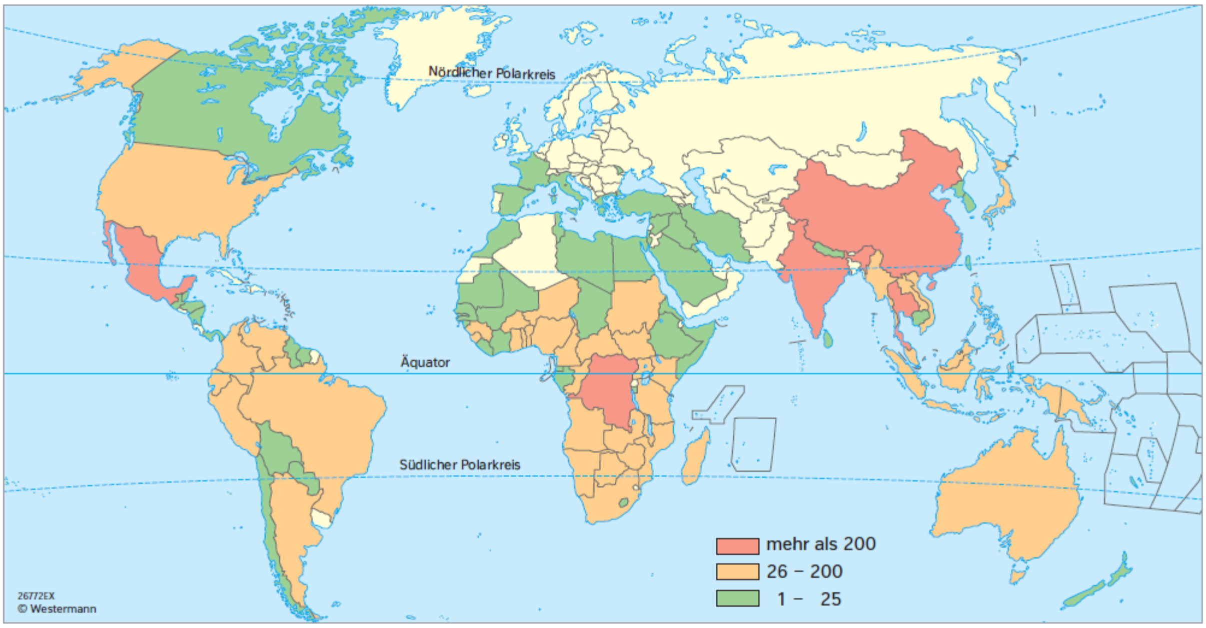

Material 2: Weltkarte der Verbreitung vom Menschen verzehrter Insektenarten.

(©Westermann nach Center for Geoinformation der Universität Wageningen, van Lammeren und Jongema 2017)

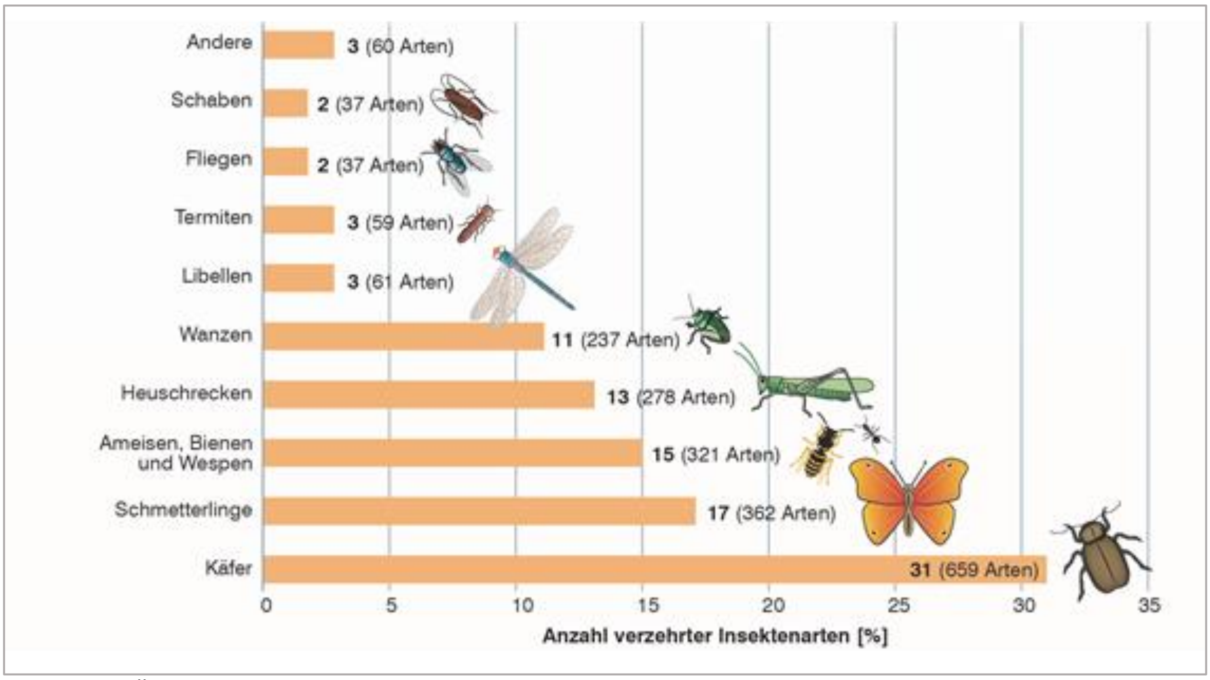

Material 3: Übersicht der weltweit vom Menschen verzehrten Insektenarten. Insgesamt haben Wissenschaftler 2.111 essbare Arten dokumentiert. Spinnen gehören biologisch gesehen nicht zu den Insekten, werden hier jedoch trotzdem aufgeführt

Eine wachsende Weltbevölkerung und die Frage danach, wie eine Welt mit mehr als neun Milliarden Menschen im Jahr 2050 bei nur begrenzten natürlichen Ressourcen ernährt werden soll, heizen die Diskussion um nachhaltige Alternativen zu herkömmlichem Fleisch als Proteinquelle an. Bei steigender Nachfrage würde die Produktion von tierischem Protein zu viele Ressourcen verbrauchen und bereits bestehende Umweltprobleme weiter verschlimmern. Die Welternährungsorganisation prognostiziert für das Jahr 2050 einen Anstieg der Nachfrage nach Fleisch von 70 bis 80 Prozent. Um diese Nachfrage zu befriedigen, wäre der Bedarf an landwirtschaftlicher Fläche, Wasser und Energie enorm. Die Produktion von Insekten für den menschlichen Verzehr weist im Vergleich zur konventionellen Tierhaltung von Hühnern, Schweinen und Rindern bei ausgewählten Nachhaltigkeitsindikatoren einige Vorteile auf (Material 4). Ob Insekten eine gesündere Alternative zu herkömmlichen Nutztieren darstellen, ist aufgrund der Vielzahl zum Verzehr geeigneter Insektenarten und einer damit einhergehenden, großen Bandbreite ihrer Nährstoffzusammensetzung nicht pauschal zu beantworten. Ihr Potenzial für eine gesunde Ernährungsweise lässt sich daher am besten an ausgewählten Insektenarten abschätzen, die schon im großen Maßstab in Europa produziert und gegessen werden, wie zum Beispiel Mehlwürmer (*Tenebrio molitor*) und Heimchen (*Acheta domesticus*) (Material 4). Aufgrund ihrer vielversprechenden Nährstoffzusammensetzung werden viele Insektenarten schon jetzt als neues „Superfood“ angepriesen, da sie eine Möglichkeit bieten, bei einer Reduktion des Fleischkonsums oder eines Fleischverzichts den Protein-, Fett- und Mineralstoffbedarf zu kompensieren. Allerdings vermehren sie sich am besten zwischen 25° - 30° Celsius, weshalb ihre Produktion – zumindest in Europa – noch relativ viel Energie benötigt.

Zudem geht von Insekten ein geringes Risiko für die Übertragung zoonotischer Krankheiten wie H1N1 (= „Schweinegrippe“) oder BSE (= „Rinderwahn“) aus. Befunde zur Übertragung von Parasiten auf den Menschen liegen bisher noch nicht vor. Trotzdem sollten Menschen mit einer Lebensmittelallergie beim Verzehr von Insekten vorsichtig sein. Es können Kreuzreaktionen bei Menschen auftreten, die allergisch auf Schalentiere (z. B. auf Garnelen oder Krebse) und Milben reagieren. Außerdem sollten Personen mit einer Pollenallergie auf den Verzehr von Bienenlarven verzichten, da diese Pollen enthalten können.

INFOBOX

Was ist eigentlich *nachhaltig*?

Das Aktionsprogramm **Agenda 21** wurde von 172 Staaten auf der **Konferenz für Umwelt und Entwicklung der Vereinten Nationen** (UNCED) 1992 in Rio de Janeiro beschlossen und setzt Leitlinien für das 21. Jahrhundert, vor allem zur **nachhaltigen Entwicklung**. An dieser Konferenz nahmen neben Regierungsvertretern auch viele nichtstaatliche Organisationen teil. Seit 2016 ist die **Nachfolgeagenda 2030** in Kraft getreten. Bereits 1987 wurde das Prinzip des nachhaltigen Handelns so beschrieben: „Nachhaltige Entwicklung ist eine Entwicklung, die die Bedürfnisse der **Gegenwart** befriedigt, ohne zu riskieren, dass **künftige Generationen** ihre eigenen **Bedürfnisse** nicht befriedigen können.“ Die Bundesregierung Deutschlands ergänzt zudem: „Der Gedanke der Nachhaltigkeit verbindet **wirtschaftliche** Leistungsfähigkeit mit **ökologischer** Verantwortung und **sozialer** Gerechtigkeit. Diese drei Ziele bedingen einander. Denn auf Dauer ist kein Wirtschaftswachstum vorstellbar, das auf Raubbau an der Natur oder auf sozialen Ungerechtigkeiten beruht“ (Bundesregierung, 2008). Verfolgt werden sollte daher auch die oben bereits erwähnte **Intra- und Intergenerationale Gerechtigkeit** und damit verbundene Chancengleichheit.

Lena Szczepanski – Osnabrück University – Department of Biology Didactics
Email: lena.szczepanski@uni-osnabrueck.de

	Mehlwurm 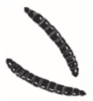	Heimchen 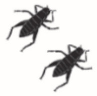	Huhn 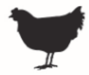	Schwein 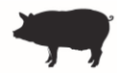	Rind 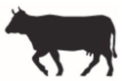
**Energiewerte und Proteingehalt**					
Energiewert [kcal/ 100g essbarer Anteil]	554	455	145	106	121
Proteingehalt [g/ 100g essbarer Anteil]	49,1	66,6	22,2	22,0	21,2
Fettgehalt [g/ 100g essbarer Anteil]	35	22,1	6,2	2,0	4,0
**Mineralstoffe [mg/ 100g essbarer Anteil]**					
Eisen	2,1	6,3	1,1	–	2,3
Calcium	16,9	132,1	14,0	2,0	3,0
Phosphor	285,0	957,8	212,0	173,0	164,0
Magnesium	80,1	109,4	-	-	22,0
**Nachhaltigkeitsindikatoren der Produktion**					
Essbarer Anteil [%]	100	80	55	55	40
Futterverwertung [kg Futter/ kg Körpermasse]	2,2	2,1	4,5	9,1	25
Energieverbrauch [MJ/ kg]	33,7	-	80-152	95-236	177-273
CO_2-Äquivalente [g/ kg Massenzuwachs]	8	2	-	1130	2850
NH_3 [mg/ Tag/ kg Massenzuwachs]	1	142	-	1920	-
Landnutzung [m²/ kg essbarer Tiermasse]	18	-	42-52	47-64	144-258
Wasserverbrauch [L/ kg essbarer Tiermasse]	4341	2000	34	57	112
Preis [€/kg]	117,80	248,75	10,0	9,00	49,90

Material 4: Energiewerte und Nährstoffzusammensetzung sowie ausgewählte Nachhaltigkeits-indikatoren zur Produktion von Mehlwürmern und Heimchen im Vergleich mit konventionellen Nutztieren.

Zusätzliche Informationen zur Interpretation der Tabelle

Beim Huhn beziehen sich die Werte auf 100g Brustfleisch, bei Schwein und Rind auf 100g Filetstücke. Bei den Insekten beziehen sich alle Werte auf unverarbeitete und rohe Tiere. Futterverwertung = Futter-Input in kg, den man benötigt, um 1kg an essbarer Tiermasse zu produzieren. CO_2-Äquivalent = *Global Warming Potential* (GWP) = Treibhauspotenzial: Maßzahl für den relativen Beitrag zum Treibhauseffekt. Energieverbrauch in MJ zur Produktion von 1 kg essbarer Tiermasse (1 MJ = 1 Megajoule = 239.006 kcal) Mit „–“ markierte Stellen in der Tabelle bedeuten, dass bisher keine Daten zu dem jeweiligen Indikator ermittelt wurden.) Die Preise für das Fleisch (Januar 2019) stammen von REWE®-Online und für die Insekten von Protifarm®.

Aufgaben

(1) Erkläre den Begriff „nachhaltig“ im Zusammenhang mit Ernährung und nenne weitere Beispiele, bei denen Nachhaltigkeit eine Rolle spielt.

(2) Fasse die Vor- und Nachteile von insektenbasierten Nahrungsmitteln tabellarisch zusammen.

Lena Szczepanski – Osnabrück University – Department of Biology Didactics
Email: lena.szczepanski@uni-osnabrueck.de

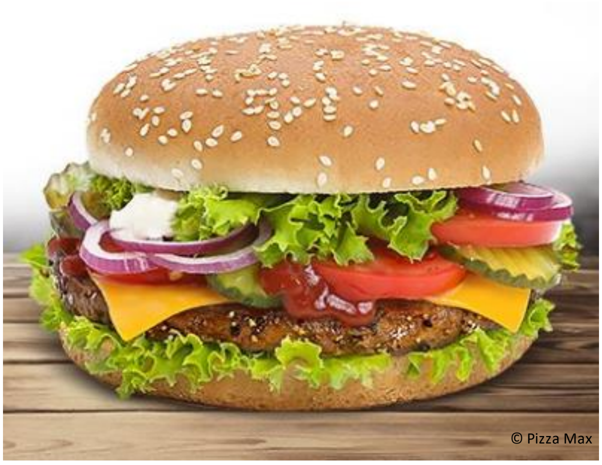

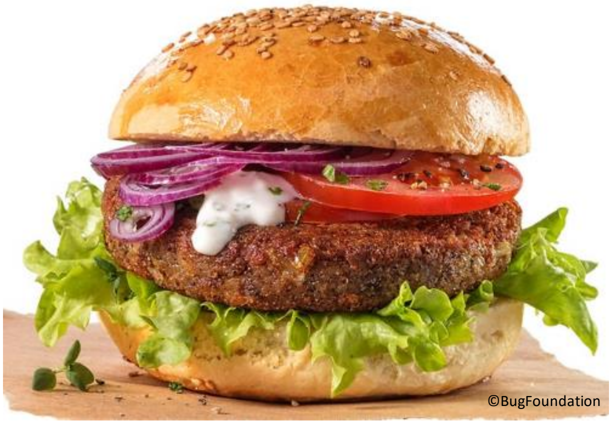

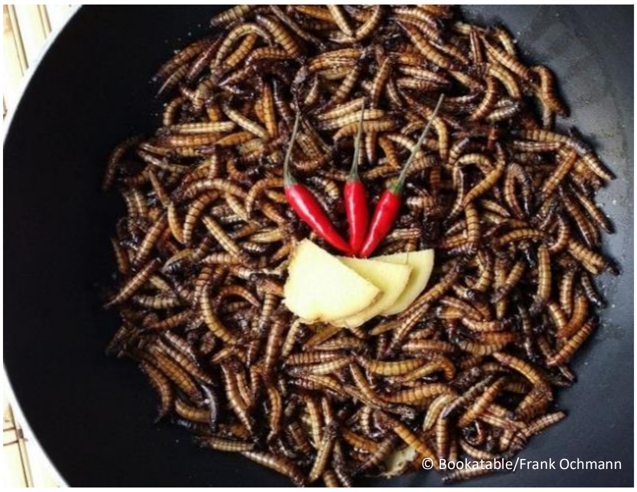

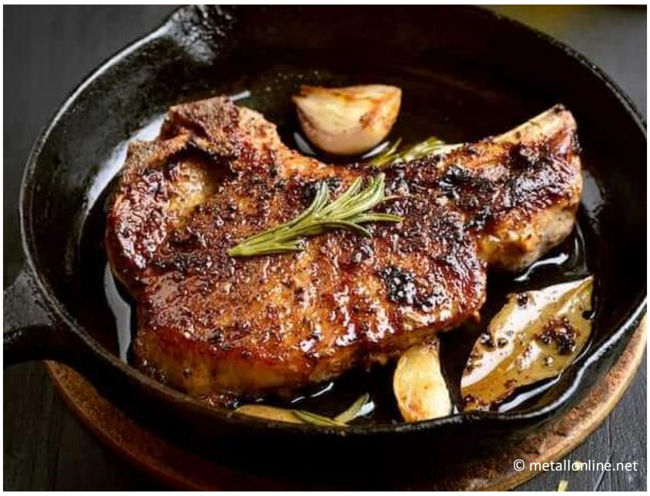

Lena Szczepanski – Osnabrück University – Department of Biology Didactics
Email: lena.szczepanski@uni-osnabrueck.de

Nach Schätzungen der Vereinten Nationen wird die Weltbevölkerung bis zum Jahr 2050 von heute etwa 7,6 Milliarden auf 9,2 Milliarden Menschen ansteigen. Durch steigende Einkommen und eine wachsende Mittelschicht - gerade in Entwicklungsländern - wird aufgrund der zunehmenden Kaufkraft auch die Nachfrage nach Lebensmitteln und insbesondere nach Fleisch, als tierische Proteinquelle, ansteigen (Material 5). Um den Hunger nach Fleisch zu stillen, werden allein in Deutschland schon heute täglich (!) 2.160.787 Tiere geschlachtet.

	1965	1975	1985	1995	1998	2015	2030	2050
Welt	24,2	27,4	30,7	34,6	36,4	41,3	45,3	49
Industrieländer	61,5	73,5	80,7	86,2	88,2	95,7	100,1	-
Schwellenländer	42,5	60,0	65,8	50,5	46,2	53,8	60,7	-
Entwicklungsländer	10,2	11,4	15,5	22,7	25,5	31,6	36,7	42
Sub-Sahara Afrika	9,9	9,6	10,2	9,3	9,4	10,9	13,4	16,0
Naher Osten/Nord Afrika	11,9	13,8	20,4	19,7	21,2	28,6	35,0	38,5
Lateinamerika (mit Karibik)	31,7	35,6	39,7	50,1	53,8	65,3	76,6	84,0
Süd-Asien (mit Indien)	3,9	3,9	4,4	5,4	5,3	7,6	11,7	18,0
Ost-Asien	8,7	10,0	16,9	31,7	37,7	50,0	58,5	71,1

Material 5: Entwicklung des weltweiten Fleischkonsums (kg/pro Kopf/Jahr) (inkl. Schaf-, Ziegen-, Geflügel-, Schweine- und Rindfleisch) (Quelle: Alexandratos und Bruinsma, 2012).

Die industrielle Tierhaltung ist mit Abstand der größte Verursacher von Landnutzungsänderungen: Insgesamt werden weltweit 70% aller landwirtschaftlichen Flächen und 30% der gesamten Landfläche unseres Planeten als Weideflächen und zur Futtermittelproduktion genutzt. Diese Landnutzungsänderungen werden als Hauptursache für den Verlust der Artenvielfalt angesehen.

Die Nutztierhaltung trägt auch maßgeblich zur Emission von Treibhausgasen bei, wie z.B. von Methan (CH_4) oder Lachgas (N_2O). Methan hat ein 23-fach so hohes, Lachgas sogar ein 296-fach so hohes Treibhausgaspotenzial wie Kohlenstoffdioxid (CO_2). Insgesamt ist die Nutztierhaltung verantwortlich für 37% der vom Menschen verursachten Methan- und 65% der anthropogenen Lachgas-Emissionen. Damit ist der Tierhaltungssektor verantwortlich für 18% der globalen Treibhausgasemission und trägt wesentlich zum Klimawandel bei, sogar mehr als der Transportsektor. Methan entsteht vor allem während des Verdauungsvorgangs bei Wiederkäuern (z.B. Rinder und Schafe) sowie bei der Lagerung von Wirtschaftsdünger (Festmist und Gülle) und wird spätestens bei der Ausbringung auf die Felder freigesetzt. Hierdurch kommt es auch zu einer übermäßigen Belastung von biogeochemischen Stoffkreisläufen mit Stickstoff- und Phosphorverbindungen.

Zudem sind Nutztierbestände die Hauptquelle für 64% der anthropogenen Ammoniak-Emissionen (NH_3). Ammoniak ist ein Luftschadstoff, der bei der Schweinehaltung direkt im Stall entsteht. Er reagiert in der Atmosphäre mit anderen Stoffen und trägt als saurer Regen erheblich zur Versäuerung und Eutrophierung (Nährstoffanreicherung) unserer Land- und Wasserökosysteme bei.

Außerdem macht der Tierhaltungssektor mehr als 8% des weltweiten Wasserverbrauchs des Menschen aus, und trägt so zu einer Übernutzung unserer Süßwasserressourcen bei.

Quellen: Autorentext nach Campbell u.a., 2017; Fiebelkorn, 2017; Steinfeld u.a., 2006

Aufgaben

(1) Vergleiche die Entwicklungen des Fleischkonsums zwischen Industrie- und Entwicklungsländern mithilfe der Daten aus Material 5, indem du ein Liniendiagramm erstellst. Fasse die wesentlichen Trends in eigenen Worten zusammen.

(2) Die Produktion von Fleisch hat viele negative Folgen für die Umwelt. Beschreibe drei der Auswirkungen in eigenen Worten.

Lena Szczepanski – Osnabrück University – Department of Biology Didactics
Email: lena.szczepanski@uni-osnabrueck.de

Forscher gehen davon aus, dass derzeit 2.111 verschiedene Insektenarten von über 2 Milliarden Menschen in mehr als 130 Ländern gegessen werden (AB1, Material 2 und 3). In vielen subtropischen und tropischen Entwicklungsländern gehören sie zum traditionellen Speiseplan und werden häufig noch wild gesammelt oder in Privathaushalten gezüchtet.
In Europa wird derzeit eine moderne Industrie zur Herstellung von Insekten aufgebaut (Material 6). Einer der heute schon im großen Maßstab für den menschlichen Verzehr produzierten Insekten sind „Mehlwürmer". Bei diesen handelt es sich im eigentlichen Sinne nicht um „Würmer", sondern um die Larven des Mehlkäfers (*Tenebrio molitor*).
Bei der einfachsten Art die Larven zu produzieren, gibt man die Eier des Mehlkäfers in eine Kiste mit Substrat (z.B. Weizenkleie und Karotten) und wartet bis die Larven auf die gewünschte Größe gewachsen sind. Nach der Trennung der Larven vom Substrat, geht ein Großteil der Larven in die Lebensmittelproduktion (ca. 95%), ein kleiner Teil wird für die Zucht neuer Mehlwürmer genutzt (ca. 5%). Für die Weiterverarbeitung werden die Larven zunächst gereinigt und getrocknet. Je nach gewünschtem Endprodukt schließen sich dann noch verschiedene Prozesse, wie z.B. das Zerkleinern oder Pressen der Larven an (Material 7). Bereits seit dem 1. Januar 2018 werden in Deutschland Nahrungsmittel aus Insekten, z.B. in Form von Pasta oder Burgern, im Supermarkt verkauft.
Aus tierethischer und ökologischer Perspektive spricht vieles für den Verzehr von Insekten, gerade im Vergleich zum Fleischkonsum. Eine allgemeingültige Aussage, ob Insekten nachhaltiger als unsere konventionellen Nutztiere produziert werden können, ist aufgrund der großen Anzahl verschiedener Insekten allerdings schwierig. Am Beispiel von Mehlwürmern wird aber deutlich, dass sie gegenüber der Produktion von konventionellen Nutztieren, in vielen Punkten nachhaltiger produziert werden können (Material 4). So benötigen sie z.B. weniger Fläche und Wasser als konventionelle Nutztiere. Allerdings vermehren sie sich am besten zwischen 25° - 30° Celsius, weshalb ihre Produktion – zumindest in Europa – noch relativ viel Energie benötigt.

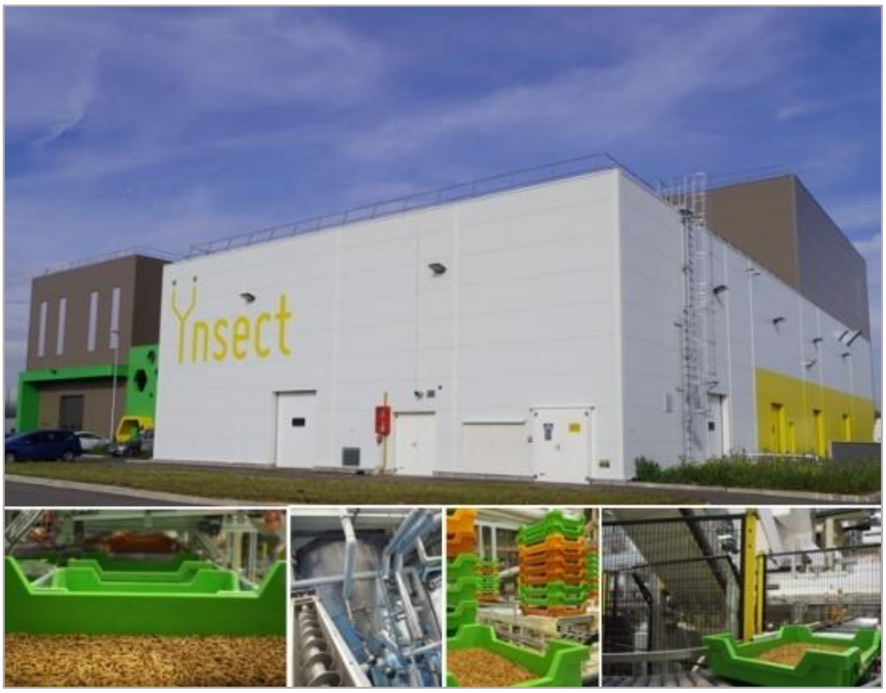

Material 6: Einblicke in eine Produktionsstätte zur Herstellung von Insekten zum menschlichen Verzehr
(© www.ynsect.com)

Lena Szczepanski – Osnabrück University – Department of Biology Didactics
Email: lena.szczepanski@uni-osnabrueck.de

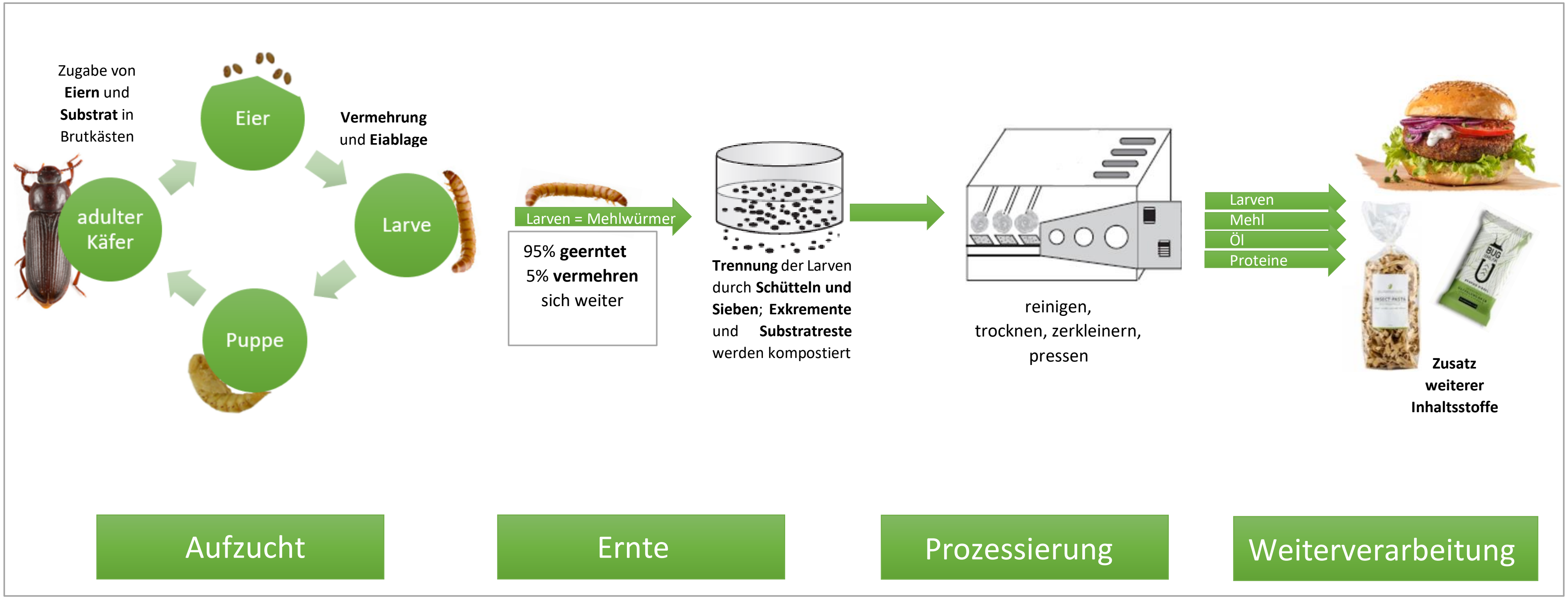

Material 7: Züchtung und Verarbeitung von Mehlwürmern zur Nahrungsmittelproduktion
© Westermann, BugFoundation, Kühne & Preißel (JKI), plumento-foods.com, bat-rodents.eu, bug-break.com, imago-insects.com

Aufgaben

(1) Beschreibe das Herstellungsverfahren von Mehlwürmern (Material 6) in eigenen Worten.

Lena Szczepanski – Osnabrück University – Department of Biology Didactics
Email: lena.szczepanski@uni-osnabrueck.de

Kriterien	Gewichtung (1 bis 3)	Fleisch (Schwein)		Insekten (Mehlwürmer)	
		Punkte	Wert	Punkte	Wert
**Preis**	2	4	2 x 4 = 8	1	2 x 1 = 2
**Summe**					

Aufgabe

Führe mit Hilfe der Tabelle und den Informationen in den Materialien (AB1-4) eine „Explizite Bewertung“ zu folgender Fragestellung durch: Welche der zwei Optionen (1) Schweinefleisch oder (2) Insekten (Mehlwürmer) bietet deiner Meinung nach eine Möglichkeit, die Menschheit auf möglichst nachhaltige Art und Weise mit tierischen Proteinen zu versorgen?

Für die Bewertung gehst du folgendermaßen vor:

(1) Schreibe mithilfe der Materialien (AB 1-4) verschiedene Kriterien auf (linke Spalte). Du kannst auch eigene Kriterien hinzufügen.

(2) Gewichte deine Kriterien (1 = unwichtig, 2 = wichtig, 3 = sehr wichtig).

(3) Nun erfolgt eine systematische Bewertung der einzelnen Kriterien. Pro Kriterium stehen dir hierfür insgesamt vier Punkte zur Verfügung. Diese werden auf die zwei Optionen (Fleisch & Insekten) verteilt.

(4) Wenn du alle Kriterien bewertet hast, errechnest du den endgültigen „Wert“ durch einfache Multiplikation. Am Ende rechnest du die Werte zusammen. Die Summe gibt an, welche Option - rein rechnerisch (!) - die nachhaltigste Wahl darstellen würde.
Ein Beispiel für die Bewertung des Kriteriums „Preis“ findest du in der Tabelle. Du kannst das Kriterium „Preis“ natürlich auch nutzen und gegebenenfalls auch anders bewerten.

Lena Szczepanski – Osnabrück University – Department of Biology Didactics
Email: lena.szczepanski@uni-osnabrueck.de

Zeit	Phase	Inhalt	Material	Sozialform
1. Doppelstunde Insektenbasierte Nahrungsmittel „Pro und Contra“, Nachhaltigkeit				
15	Fragebogen	Fragebogen ausfüllen	Fragebogen	EA
5	Einstieg	-Maikäfersuppe Rezept	PP Beamer/ OHP	UG
5	Erarbeitung I	- Text lesen „Insekten als Nahrungsmittel in DL und der Welt“	AB1	EA/ PA
10	Lösungsplanung I	- Diskutiere, aus welchen Gründen es zu der Trendwende gekommen sein könnte, dass in Deutschland seit 2018 Insekten als Nahrungsmittel zugelassen sind.<br>- Hypothesensammlung	Beamer bzw. Tafel	UG
25	Erarbeitung II	1) Recherchiere, welche Nahrungsmittel aus Insekten in DL schon erhältlich sind.<br>2) Lese den Text „Nahrungsmittel aus Insekten – nachhaltig und gesund?“ (AB2).<br>3) Erkläre den Begriff „nachhaltig“ im Zusammenhang mit Ernährung und nenne weitere Beispiele, bei denen Nachhaltigkeit eine Rolle spielt.<br>4) Fasse die Vor- und Nachteile von insektenbasierten Nahrungsmitteln tabellarisch zusammen.	PC/ Handy<br><br>Aufgabenstellung über Beamer<br>AB2	PA
25	Sicherung II	- Beispielnahrungsmittel besprechen<br>- Tafelbild zur „Nachhaltigkeit“ festhalten<br>- Tabelle Vor- und Nachteile ausfüllen (Beamer/ Tafel)<br>- Ausgangsfrage Trendwende – Antwort auf Stundenfrage diskutieren	PC Beamer<br>Tafel	UG

Lena Szczepanski – Osnabrück University – Department of Biology Didactics
Email: lena.szczepanski@uni-osnabrueck.de

Zeit	Phase	Inhalt	Material	Sozialform
2. Doppelstunde explizites Bewerten, systematischer Umgang mit Entscheidungssituationen				
10	Einstieg	Bilder zeigen und darüber abstimmen, wer zu welchem Nahrungsmittel tendieren würde. (intuitives Bewerten)<br><br>*Heute wollen wir ein bisschen tiefer in die Materie einsteigen und einen direkten Vergleich von Insekten und Schweinefleisch anstellen.*<br>⇨ AB5 Tabelle als Stundenziel vorstellen	Beamer/ OHP<br><br>Bilder Burger und Essen in Pfanne	UG
40	Erarbeitung I	Aufgaben auf AB3-4<br>AB5:<br>1. Zehn Kriterien in Tabelle (mithilfe von AB1-4)<br>2. Kriteriengewichtung<br>3. Systematisches Bewerten (1-4 Punkte pro Kriterium)<br>5. Berechnung der „Werte“	Infos: von letzter Woche AB1 & AB2,<br><br>neue Texte (AB3-AB5)<br><br>Internet-recherche	GA
20	Sicherung I	- Meinungsbild abfragen<br>- Diskutieren (Diskrepanz intuitives Bewerten vom Beginn der Stunde vs. Ergebnis der expliziten Bewertung hinsichtlich der Nachhaltigkeit); Modellkritik (Kriterien aufstellen und gewichten auch subjektiv; Veränderung des Ergebnisses je nach Perspektive des Bewerters, z.B. Ernährungsprofessor, …)<br>- letzte Chance, Insekten zu probieren	PC<br>Tafel	SV
15	Fragebogen	Fragebogen ausfüllen	Fragebogen	EA
5	Ausklang/ Verabschiedung	Bedanken für die Teilnahme an meiner Studie. offene Diskussion über Unterricht und Entomophagie		UG

Lena Szczepanski – Osnabrück University – Department of Biology Didactics
Email: lena.szczepanski@uni-osnabrueck.de
